# The relationship between pathological brain activity and functional network connectivity in glioma patients

**DOI:** 10.1007/s11060-024-04577-7

**Published:** 2024-02-03

**Authors:** Mona L. M. Zimmermann, Lucas C. Breedt, Eduarda G. Z. Centeno, Jaap C. Reijneveld, Fernando A. N. Santos, Cornelis J. Stam, Marike R. van Lingen, Menno M. Schoonheim, Arjan Hillebrand, Linda Douw

**Affiliations:** 1 Anatomy and Neurosciences, Amsterdam UMC Location Vrije Universiteit Amsterdam, Amsterdam, The Netherlands; 2 Amsterdam Neuroscience, Amsterdam UMC Location Vrije Universiteit Amsterdam, Amsterdam, The Netherlands; 3 Cancer Center Amsterdam, Amsterdam UMC Location Vrije Universiteit Amsterdam, Amsterdam, The Netherlands; 4 Univ. Bordeaux, CNRS, IMN, UMR 5293, Bordeaux, France; 5 Department of Neurology, Stichting Epilepsie Instellingen Nederland, Heemstede, The Netherlands; 6 Dutch Institute for Emergent Phenomena (DIEP), Institute for Advanced Studies, University of Amsterdam, Amsterdam, The Netherlands; 7 Clinical Neurophysiology and MEG Center, Amsterdam UMC Location Vrije Universiteit Amsterdam, Amsterdam, The Netherlands

**Keywords:** Graph theory, Cancer neuroscience, Clinical neurophysiology, Brain tumor

## Abstract

**Purpose:**

Glioma is associated with pathologically high (peri)tumoral brain activity, which relates to faster progression. Functional connectivity is disturbed locally and throughout the entire brain, associating with symptomatology. We, therefore, investigated how local activity and network measures relate to better understand how the intricate relationship between the tumor and the rest of the brain may impact disease and symptom progression.

**Methods:**

We obtained magnetoencephalography in 84 de novo glioma patients and 61 matched healthy controls. The offset of the power spectrum, a proxy of neuronal activity, was calculated for 210 cortical regions. We calculated patients’ regional deviations in delta, theta and lower alpha network connectivity as compared to controls, using two network measures: clustering coefficient (local connectivity) and eigenvector centrality (integrative connectivity). We then tested group differences in activity and connectivity between (peri)tumoral, contralateral homologue regions, and the rest of the brain. We also correlated regional offset to connectivity.

**Results:**

As expected, patients’ (peri)tumoral activity was pathologically high, and patients showed higher clustering and lower centrality than controls. At the group-level, regionally high activity related to high clustering in controls and patients alike. However, within-patient analyses revealed negative associations between regional deviations in brain activity and clustering, such that pathologically high activity coincided with low network clustering, while regions with ‘normal’ activity levels showed high network clustering.

**Conclusion:**

Our results indicate that pathological activity and connectivity co-localize in a complex manner in glioma. This insight is relevant to our understanding of disease progression and cognitive symptomatology.

**Supplementary Information:**

The online version contains supplementary material available at 10.1007/s11060-024-04577-7.

## Introduction

Prognosis of glioma is poor, and many patients experience debilitating symptoms. Patients show differences in whole-brain neurophysiology compared to controls. Disturbances in neuronal activity and functional brain network connectivity have been found throughout the entire brain. However, it is unclear how these indices of brain functioning relate to each other, while the interplay between activity and connectivity might be essential for prognosis and patient functioning.

In preclinical studies, glioma cells reciprocally interact with their immediate neuronal environment [1, 2]. Via the formation of neuron-to-glioma synapses, neuronal spiking activity in the tumor’s proximity promotes tumor proliferation and invasion [2, 3]. Translational studies have used magnetoencephalography (MEG) as a non-invasive measurement of neuronal activity, reporting high activity around the tumor and across the tumor hemisphere as compared to controls [4]. This pathologically high activity relates to shorter progression-free survival [5, 6], underlining the clinical relevance of widespread activity for tumor growth.

Glioma patients also show different functional connectivity between brain regions compared to healthy people. Functional connectivity is the statistical dependency between activity patterns from different brain regions [7]. Network theory can be used to extract regional or whole-brain topological markers from this connectivity [8, 9]. A combination of local specialization (segregation) and overall integration is considered essential for network functioning [9]. Glioma patients show higher segregative connectivity and lower integrative connectivity in comparison to controls [10–14]. Higher functional connectivity of the tumor region associates with shorter survival [14]. Moreover, pathologically high clustering, describing the segregative properties of the network [8, 9], relates to poorer cognitive performance [13, 15–17]. These disturbances go beyond the (peri)tumoral region and are a truly global network pathology [12].

In support of the idea that the interaction between the ‘rest of the brain’ and glioma is complex and clinically important, we recently found that gliomas tend to occur in regions with intrinsically higher brain activity in controls [18]. Moreover, while most tumors seem to occur in regions with intrinsically high clustering and integrative connectivity [18–20], patients with gliomas in regions with intrinsically low clustering have more extensively different network clustering at diagnosis [12]. However, it is unclear how activity and connectivity are interrelated throughout the brain. Answering this question could help guide our thinking on tumor-brain cross-talk and its impact on disease progression and symptomatology.

Here, we investigated how these two aspects of neurophysiological functioning co-occur regionally, by collecting MEG in glioma patients and controls. Since higher local activity measured with MEG reflects more synchronous activity of large groups of neurons and potentially higher local clustering [21], and, brain regions with higher neuronal spiking activity show higher integrative connectivity in (computational) studies [22–24], we hypothesized positive correlations between activity and connectivity, at least in controls.

## Materials and methods

### Participants

Patient (preoperative) data stemmed from an ongoing study of the Amsterdam UMC location VUmc (Supplementary Table S1). Inclusion criteria were glioma of grade ≥ II [25], age > 18 years and no neuropsychiatric disorders or comorbidities of the central nervous system.

Healthy controls (HCs) came from two studies using the same MEG system and procedures [26, 27]. They were matched to patients on sex and age.

We investigated activity and network connectivity at the (peri)tumor area, its contralateral homologue, and all areas without tumor. To define the (peri)tumoral regions, tumor masks were manually drawn in [LD] on post-gadolinium T1-weighted and FLAIR anatomical images, or automatically segmented and visually checked [28]. Regions (Brainnetome atlas, BNA [29]) were considered part of the (peri)tumoral area when ≥ 12% of their volume overlapped with the tumor mask (Supplementary materials). The contralateral homologue of the (peri)tumoral area was the same tumor region(s) but in the contralateral hemisphere. Patients with bilateral tumors or tumors without regional overlap were excluded from analyses concerning the (peri)tumoral and homologue areas. The rest of the brain were all regions with 0% tumor overlap.

### Magnetoencephalography

Participants underwent 5-min eyes-closed resting-state MEG, using a 306-channel Elekta Neuromag Oy MEG system, with a sampling frequency of 1250Hz and 0.1Hz high pass and 410Hz antialiasing filters (Supplementary materials). We used cross-validation signal space separation, after which raw data were visually inspected and malfunctioning channels excluded [LD]. The signal was filtered between 0.5-45Hz. We used a 3D digitizer (Fastrak; Polhelmus) to digitize 4–5 head position indicator coils and the scalp-nose surface for co-registration of the MEG data to patients’ anatomical MRIs. A scalar beamformer implementation (Elekta Neuromag Oy, version 2.1.28) source-reconstructed broadband (0.5-45Hz) time series to the 210 BNA centroids [27, 29, 30]. We selected 15 and 8 epochs for patients and HCs, respectively (3.27s).

### Regional brain activity

The offset of the aperiodic part of the power spectrum was used as a proxy for neuronal spiking activity [31]. Power spectra were obtained using Welch’s method with a Hamming window for each epoch and cortical brain region. These were averaged over all epochs per subject to obtain one spectrum per brain region. The Fitting Oscillations & One Over F (FOOOF) toolbox was used to estimate the offset by fitting the non-oscillatory part of the power spectrum using the exponential function: L = b – log(k + Fx) (Supplementary materials) [31].

**Fig. 1:**
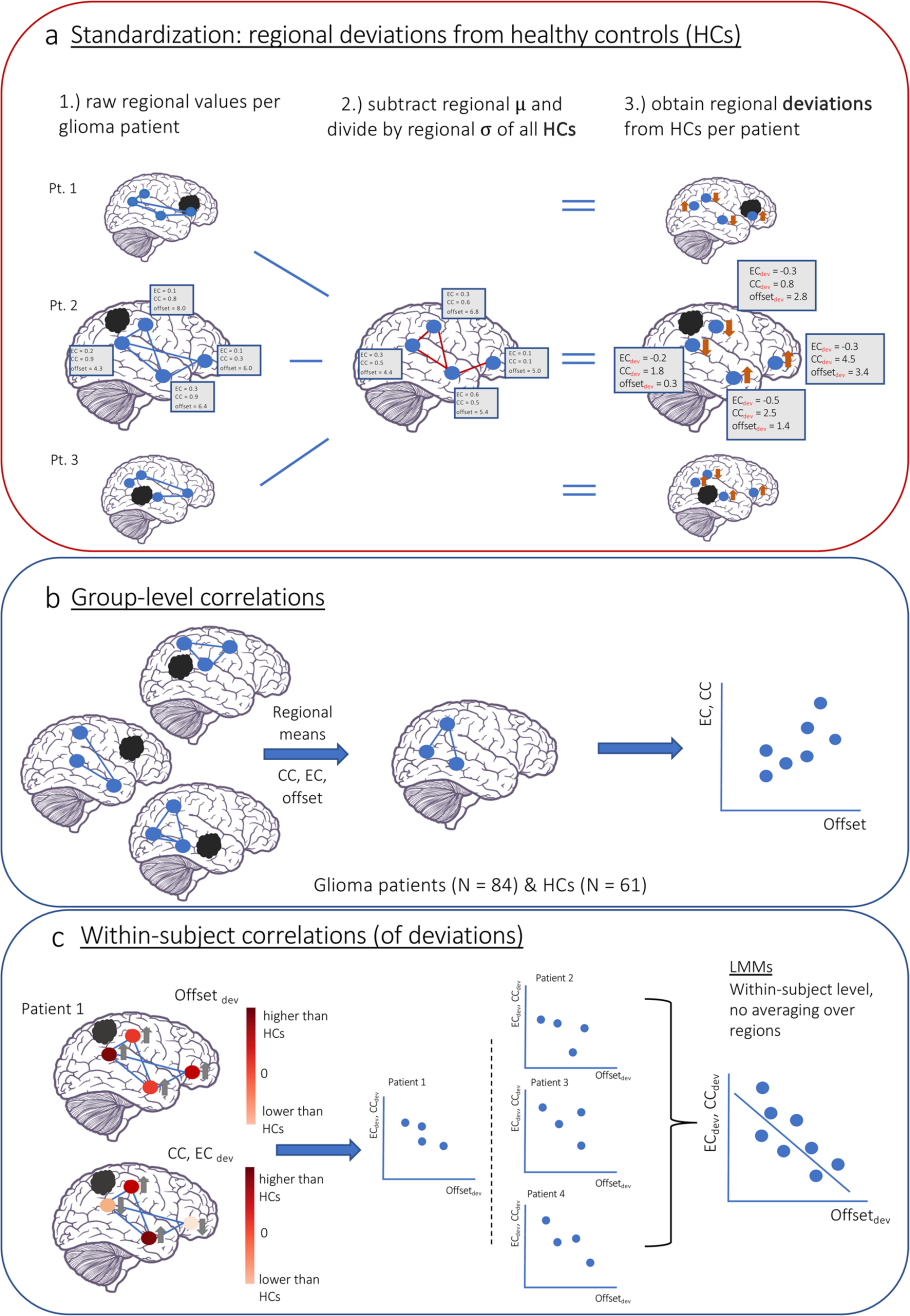
**a** Standardization procedure to obtain regional deviations. **b** Group-level analysis using regional means of the raw offset, EC and CC values for the spin-test [32] **c** Within-subject analysis with standardized values and LMMs

To obtain values representing deviations from HCs, we standardized patients’ and HCs’ regional values using the regional mean and standard deviation of HCs (hereafter referred to as ‘dev’; Fig. 1).

### Functional networks

Functional networks were constructed in Python.We employed a fast Fourier transform-based band-pass filter to every epoch per brain region. Functional networks were reconstructed for the delta (0.5-4Hz), theta (4-8Hz), and lower alpha (8-10Hz) bands [10, 17]. We used the Phase Lag Index (PLI) to calculate functional connectivity between all 210 cortical regions [13, 15, 17, 33]. PLIs were calculated per epoch and averaged over epochs per frequency band. We thresholded and binarized frequency-specific networks using a proportional threshold, keeping the strongest 20% or 30% of connections, yielding six networks per participant (Supplementary materials). We avoided constructing networks with many nodes (e.g.threshold of 10%) or highly connected networks with many false positive connections (i.e. threshold of > 30%) [34].

We calculated the regional local clustering coefficient (CC) and eigenvector centrality (EC) using the Networkx package [35]. The local CC denotes the number of triangles formed between neighboring regions, representing segregative, ‘local’ connectivity [9]. EC reflects the integrative properties of a node. It denotes the number of connections and connections of a nodes’ neighbors and neighbor’s neighbors etc. [36].

All CC and EC values were standardized, representing deviation from controls (CC_dev_, EC_dev_).

### Statistical analysis

To test matching between patients and HCs, we used Mann–Whitney U and Chi-square tests.

Differences in offset_dev_, local CC_dev_ and EC_dev_ between patients’ (peri)tumoral, homologue and rest of the brain values and HCs whole-brain values_dev_ were calculated using the Mann–Whitney U test. To test whether (peri)tumoral and contralateral homologue areas differed within patients, Wilcoxon signed-rank tests were used. Tests were performed for two network densities and three frequency bands.

To explore the group-level relationship between regional activity and functional network connectivity (Fig. 1) we averaged raw offset, EC and CC values for every region over all participants per group. We correlated offset with EC and CC using the spin test with a Pearson’s correlation implementation (5000 permutations) [32].

For within-subject effects (Fig. 1), we used standardized values and linear mixed models (LMMs) to handle within-subject dependencies between regions (Supplementary materials for alternative approach). We fitted a model with offset_dev_ as independent variable and CC_dev_ and EC_dev_ as predictors and a random intercept for participants (Python, statsmodels). We fitted a separate model for every frequency band, density and group. In another six models, group differences were tested through interaction terms. We reran this analysis using standardized values to obtain effect size metrics. In patients, all analyses focused on the rest of the brain.

P-values were adjusted for multiple comparisons (across frequency bands and densities, false discovery rate, FDR [37]) and deemed significant at *p*_FDR_ < 0.05. Results replicated for both network densities (20%, 30%) were deemed robust.

## Results

### Participant characteristics

84 glioma patients and 61 HCs, similar in sex (*p* = 0.053) and age (*p* = 0.115), were included (Table 1).

**Table 1:** Participant characteristics

Characteristics	Healthy controls (*N* = 61)	Glioma patients (*N* = 84)	*p*
Age (mean (SD))	48.03 (9.62)	45.68 (15.23)	0.115
Sex (number of females (males))	27 (34)	23 (61)	0.053
Tumor WHO grade (II/III/IV)	NA	37/17/30	
Tumor histology (GBM/A/O/NA)	NA	30/30/23/1	
Tumor volume, corrected for headsize (mean ml (SD))	NA	39.8 (38.0)	
Tumor side (left/right/bilateral)	NA	49/31/4	
IDH-mutant, 1p/19q non-codeleted glioma (number (%))	NA	28 (33.3)	
IDH-mutant, 1p/19q-codeleted glioma (number (%))	NA	17 (20.2)	
IDH-wildtype glioblastoma (number (%))	NA	30 (35.7)	
Unknown molecular subtype (number (%))	NA	9 (10.7)	
Epilepsy (yes (no))	NA	70 (14)	
KPS (median (range) / NA)	NA	100 (50—100) / 11	

Tumor histology is based on the 2007 and 2021 WHO classification of human brain tumors [25, 38]. IDH-mutation status and codeletion status were identified using the 2021 WHO classification and extrapolated for the subjects recruited before 2016.

*SD* Standard Deviation, *GBM* Glioblastoma, *A* Astrocytoma, *O* Oligodendroglioma, *NA* Not available

### Higher activity and clustering in patients

**Fig. 2:**
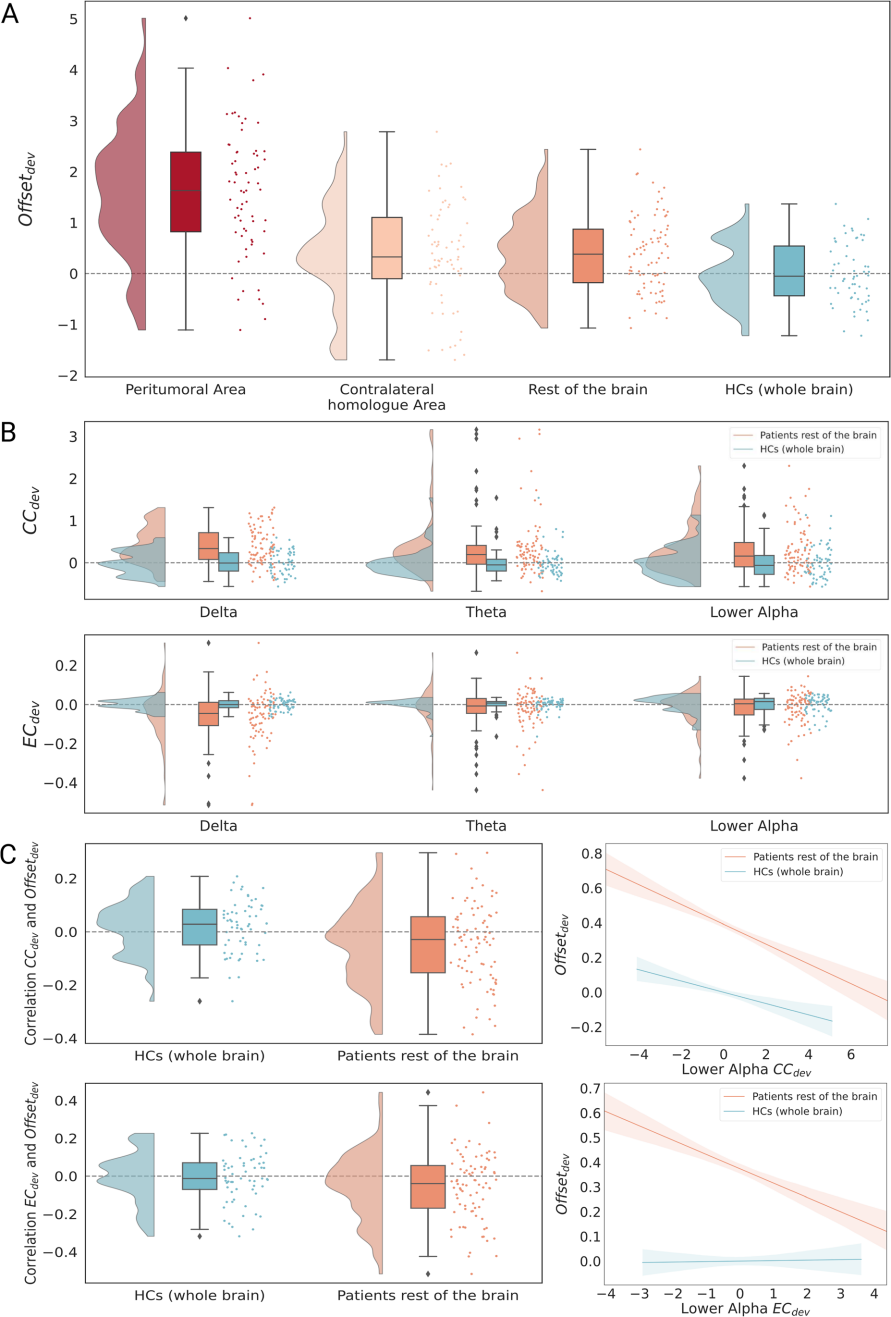
**a** Offset in (peri)tumoral, homologue and rest of the brain areas in glioma patients and the whole brain of HCs. **b** Delta, theta and lower alpha band CC_dev_ (higher panel) and EC_dev_ (lower panel) in patients (rest of the brain) and HCs (30% density of network). **c** Within-subject relations between offset_dev_ and lower alpha band CC_dev_ (higher panel) and EC_dev_ (lower panel; rest of the brain area; 30% density of network; plots created with [39])

Patients’ offset_dev_ was significantly higher than controls’ in all areas: the (peri)tumoral area (*mean* = 1.559, *SD* = 1.513, *U* = 3554, *p*_*FDR*_ =  < *0.001*), contralateral homologue area (*mean* = 0.373, *SD* = 1.168, *U* = 2616, *p*_*FDR*_ = 0.006) and rest of the brain (*mean* = 0.375, *SD* = 1.269, *U* = 3319, *p*_*FDR*_ = 0.004), suggesting that brain activity is pathologically high throughout the brain in glioma (Fig. 2a). (Peri)tumoral offset_dev_ was higher than its contralateral homologue (*Z* = 133, *p* < 0.001).

Compared to HCs, CC_dev_ was higher (peri)tumorally (delta band), in the contralateral homologue (delta, lower alpha band) and the rest of the brain (all bands, Table 2, Supplementary Table S2 for 30% density, Fig. 2b). Delta band EC_dev_ was lower in patients in the rest of the brain (Table 2, Fig. 2b). These results suggest that clustering is globally higher in patients, while integrative connectivity is lower in the non-tumoral area. CC_dev_ and EC_dev_ did not differ between the (peri)tumoral area and its homologue within patients (Supplementary Table S4, Fig. 2b).

**Table 2:** Network characteristics (density 20%) in the investigated areas of patients and their comparison to whole brain characteristics of HCs

Measure	Delta			Theta			Lower Alpha		
	20%			20%			20%		
	mean (SD)	*U*	*p* (*p*_*FDR*_*)*	mean (SD)	*U*	*p* *(p*_*FDR*_*)*	mean (SD)	*U*	*p* *(p*_*FDR*_*)*
Clustering Coefficient									
(Peri)tumoral Area	0.370 (1.179)	2910	< 0.001 (< 0.001**)	0.257 (1.447)	2254	0.316 (0.507)	0.129 (1.323)	2232	0.368 (0.507)
Contralateral Homologue Area	0.276 (1.137)	2817	< 0.001 (< 0.001**)	0.289 (1.301)	2167	0.557 (0.557)	0.233 (1.227)	2619	0.006 (0.009*)
Rest of the brain	0.327 (1.225)	3857	< 0.001 (< 0.001**)	0.324 (1.410)	3634	< 0.001 (< 0.001**)	0.316 (1.363)	3424	< 0.001 (< 0.001**)
HCs	0 (0.992)			0 (0.992)			0 (0.992)		
Eigenvector Centrality									
(Peri)tumoral Area	0.139 (1.313)	2288	0.244 (0.717)	-0.010 (1.114)	1967	0.717 (0.717)	-0.092 (1.044)	1674	0.08 (0.470)
Contralateral Homologue Area	0.037 (1.059)	2121	0.713 (0.966)	0.058 (1.109)	2167	0.557 (0.966)	-0.031 (1.079)	1983	0.775 (0.966)
Rest of the brain	-0.061 (1.089)	1663	< 0.001 (< 0.001**)	-0.032 (1.118)	2163	0.110 (0.166)	-0.033 (1.139)	2093	0.061 (0.121)
HCs	0 (0.992)			0 (0.992)			0 (0.992)		

For network characteristics *p*-values were corrected for the different frequencies and densities. The means of the measures were calculated with the values standardized on the regional means and SD of HCs (dev). Therefore, the mean of HCs is 0 and the SD around 1. Here density 20% is shown. Results for density 30% are similar and can be found in the supplementary Table S2

*HCs* Healthy controls; *SD* Standard Deviation; *U* U statistic of the Mann–Whitney U test; *p*_*FDR*_ False Discovery Rate adjusted *p*-value

* indicates *p* < 0.05, ** indicates *p* < 0.001

A post-hoc test revealed similar profiles for the different glioma subtypes (Supplementary Tables S5-S7).

### Positive group-level regional correlations

Raw offset values related positively to clustering across frequencies in HCs and patients, while it related positively to theta band EC in HCs, but not patients (Supplementary Table S8).

### Negative within-patient regional correlations

Within-patients, regional offset_dev_ related negatively to regional lower alpha band CC_dev_ in the rest of the brain of patients (Table 3, Fig. 2C, Supplementary S3). This relationship differed significantly from that in HCs (Supplementary Table S9), where no significant associations were found for the lower alpha band (Supplementary Table S10). HCs showed a positive relationship between offset_dev_ and CC_dev_ in the delta band (Supplementary Table S11).

**Table 3:** Linear mixed model with offset_dev_ as dependent and EC_dev_ and CC_dev_ as independent variables for the rest of the brain of patients

Frequency, Density	Variable	Coefficient [CI]	Std coefficient (beta)	*Z*	*p*	*p*_*FDR*_
Delta						
20%	Intercept	0.383 [0.225, 0.542]		4.734	< 0.001	
	EC_dev_	0.005 [-0.010, 0.020]	0.004	0.681	0.496	0.541
	CC_dev_	0.011 [-0.003, 0.025]	0.011	1.541	0.123	0.211
30%	Intercept	0.381 [0.223, 0.540]		4.708	< 0.001	
	EC_dev_	-0.001[-0.016, 0.013]	-0.001	-0.189	0.850	0.850
	CC_dev_	0.013[-0.001, 0.027]	0.013	1.85	0.064	0.150
Theta						
20%	Intercept	0.385 [0.226, 0.544]		4.75	< 0.001	
	EC_dev_	0.013 [-0.001, 0.028]	0.012	1.778	0.075	0.150
	CC_dev_	0.007 [-0.007, 0.020]	0.007	0.984	0.325	0.390
30%	Intercept	0.384 [0.225, 0.543]		4.739	< 0.001	
	EC_dev_	0.011 [-0.004, 0.025]	0.009	1.436	0.151	0.227
	CC_dev_	0.008 [-0.006, 0.022]	0.008	1.111	0.267	0.356
Lower Alpha						
20%	Intercept	0.392 [0.234, 0.551]		4.845	< 0.001	
	EC_dev_	-0.063 [-0.077, -0.048]	-0.056	-8.349	< 0.001	< 0.001**
	CC_dev_	-0.025 [-0.039, -0.012]	-0.027	-3.636	< 0.001	< 0.001**
30%	Intercept	0.398 [0.240, 0.557]		4.922	< 0.001	
	EC_dev_	-0.056 [-0.071, -0.042]	-0.05	-7.564	< 0.001	< 0.001**
	CC_dev_	-0.044 [-0.058, -0.030]	-0.044	-6.103	< 0.001	< 0.001**

* indicates *p* < 0.05, ** indicates *p* < 0.001; A random intercept was fitted for participants; *CI* Confidence interval for coefficient; *Std* Standardized; *p*_*FDR*_ False Discovery Rate adjusted *p*-value. The *p*-values were corrected for the different frequency bands and densities. Only the independent variables were included in this correction

These results counterintuitively indicate that in patients, regionally, pathologically high offset associates with lower deviating CC, even though our previous results established pathologically high offset and CC in patients throughout the brain. As can be seen in Fig. 2C, offset_dev_ values that were more similar to HCs were associated with pathologically high CC_dev_.

Similarly, offset_dev_ related negatively to lower alpha band EC_dev_ (Table 3, Fig. 2C). Again, there was a significant difference between patients and HCs in this relationship (Supplementary Table S9), with HCs not showing an association between offset_dev_ and EC_dev_ in the lower alpha band (Supplementary Table S10). HCs showed a positive relationship between offset_dev_ and EC_dev_ in the delta band (Supplementary Table S10), which differed significantly from patients (Supplementary Table S9).

Using Pearson correlations yielded similar results as the LMMs (Supplementary Table S11).

The negative relationships were predominantly present in IDH-wildtype glioblastomas (between CC_dev_, EC_dev_ and offset_dev_) and IDH-mutant, 1p/19q codeleted gliomas (between EC_dev_ and offset_dev_, Supplementary Tables S12-S13). Local (peri)tumoral activity did not drive these correlations (Supplementary Table S14). The post-hoc covariates handedness, tumor lateralization and tumor in dominant hemisphere were not significant (Supplementary Tables S18-S20).

Reanalysis with the cortical Automated Anatomical Labeling atlas [40] yielded similar directions and sizes of effects (Supplementary Tables S15-S17). However, lower alpha band clustering only stayed significant for 30% density in the LMMs. This relationship significantly differed from HCs for CC_dev_ (30% density), but not for EC_dev_. The Pearson’s correlational analyses were not significant.

## Discussion

We investigated how regional brain activity and functional network connectivity relate to each other in glioma patients and healthy subjects. We found that glioma patients had higher brain activity and network clustering but lower regional centrality than controls, corroborating previous MEG and functional MRI studies [10–13, 16, 17, 41–43]. Interestingly, regions marked by pathologically high brain activity typically showed very low network clustering compared to controls, while regions with regular brain activity had very high network clustering.

As expected, brain activity was higher around the tumor, which aligns with others’ and our previous work [2, 4]. We may speculate that around the tumor, heightened activity is driven by direct and indirect reciprocal neuron-glioma cell interactions [1, 2]. The mechanisms leading to heightened activity further away from the tumor, as we find here and in previous work [2, 4], remain elusive. Do invasive glioma cells form neuron-glioma synapses far away from the tumor? Does high activity in the (peri)tumoral area ‘spread’ to other brain areas? Or are oncometabolites stemming from the IDH-mutant enzyme responsible for higher excitability, acting in a similar way as glutamate [44]?

Similarly, what mechanisms could be responsible for the widespread network disturbances in segregative and integrative connectivity? We could speculate that the growing tumor initially has a local impact on the functional network, which affects the network further away, potentially through cascadic network failure [45]. Also, a recent study finds that glioblastomas remodel functional neural circuits and this associates with worse prognosis and cognition [14]. Alternatively, *invasive* glioma cells might affect the functional network topology throughout the brain. Such cells infiltrate the surrounding brain via white matter tracts, blood vessels, and microtubules. There, they adhere to other cells, such as neurons [46]. Potentially, the invasive cells impact the functioning of neuronal cell populations further away from the tumor mass, impacting functional network dynamics. Especially clustering is typically a local network process emerging from the communication between (functionally) close groups of neurons [21]. Such local cellular dynamics might regionally disturb this local process in regions away from the tumor. Also, the biological characteristics of regions largely determine functional brain network dynamics [47]. The biological underpinnings of the abstract graph theoretical measures may therefore give insight into their disturbances—particularly in glioma, in which we may expect pathological cellular processes. Longitudinal studies investigating these cellular processes and directly connecting them to the functional network differences are warranted.

Our findings confirm the hypothesis that higher regional brain activity generally relates to higher connectivity, as seen in our group-level results. Indeed, few studies investigated the relationship between brain activity and connectivity and found that they positively relate to each other in the healthy setting [22–24].

Interestingly, our within-patient analyses revealed a different relationship between activity and connectivity. Deviations in brain activity and lower alpha clustering related negatively, indicating that pathologically high regional brain activity went hand in hand with very low clustering within the same patient. Conversely, regions with more typical activity levels show either normal or very high clustering. This was surprising as we found brain activity and clustering to be high throughout the patients’ brains. This study was cross-sectional, so we cannot draw clear conclusions on the chronological emergence of deviations in clustering and brain activity. However, hypothetically, regions with the highest brain activity show altered connectivity patterns and they disconnect from other regions. For regions showing activity similar to HCs but very high clustering, we may postulate a protective pattern: maybe high clustering helps to maintain normal levels of activity throughout the brain by ‘breaking up’ the functional network. This scenario was posited in the epilepsy literature previously, which is relevant to the here studied glioma population, as more than 80% of patients included suffered from epilepsy. Epilepsy patients show less integration and heightened segregation of the functional network during the interictal period [48]. The epileptic zone is functionally isolated from other regions through higher connectivity within itself and lower centrality, potentially lowering susceptibility to new seizures [49]. This mechanism may play a role in glioma patients, who often suffer from epilepsy [50].

Maybe the growth rates of different tumor subtypes also play a role. Hypothetically, in slow-growing oligodendrogliomas, network deviations are present in the entire brain due to plasticity. This might explain why we observed this interesting relationship for the oligodendrogliomas but not the faster-growing astrocytomas. Counterintuitively then, the fastest-growing IDH-wildtype GBMs, showed a similar effect as the oligodendrogliomas. As this study was cross-sectional, we cannot disentangle the relevance of tumor growth rate. Also, the small-sized groups were potentially not powered for this subgroup analysis.

We similarly found a negative relationship between brain activity and centrality in the lower alpha band. This was less surprising, as we observed centrality to be lower throughout the brain in the patient group, but only for the delta band. This might indicate that regions showing the most pathologically high activity exhibit the lowest levels of centrality and vice versa. This is relevant when considering the cascadic network failure model, where central regions take over functions from lesioned regions [45]. Subsequently, the central region may fail as a network hub. Speculatively, such failing is reflected in our results: regions with the highest activity failed to be central integrators and showed the lowest centrality. Interestingly, (peri)tumoral activity is pathologically high in glioma, while glioma is known to occur most often in regions with intrinsically high activity and connectivity in controls [18–20]. Based on our cross-sectional data, we cannot disentangle whether high-activity regions in glioma were premorbidly highly active or became pathological upon glioma occurrence.

## Limitations

This study was cross-sectional and correlational, limiting conclusions on the chronology of disturbances and their development. The patient group was heterogeneous including different tumor subtypes, complicating the interpretation of results. Another limitation is the size of the effects observed. Also, specific preprocessing choices may have affected the activity measures. Future studies should explore the parameters of the FOOOF model. The same holds for choices in thresholding and binarization of the functional networks. Researchers should clearly report the preprocessing and analytical choices they make and share their data, to make the field more consistent.

## Conclusion

While brain activity and local clustering are pathologically high throughout the brain in glioma patients, regionally, these neurophysiological deviations present in a complex manner at the individual patient level. Future studies should further characterize these deviations and their development over time. Is the negative relationship strongest close to the tumor or further away e.g. in relevant cognitive networks? Can we predict patients’ clinical and cognitive trajectories using these deviations? Answering these questions may aid in uncovering how neuron-glioma interactions shape clinical functioning and prognosis. Ultimately, this may help clinicians to communicate expectations and identify patients that would benefit from clinical interventions.

### Supplementary Information

Below is the link to the electronic supplementary material.

Supplementary file1 (DOCX 135 KB)

## Data Availability

Data will be made available under reasonable request. All scripts used to analyze the data can be openly accessed on our github repository: https://github.com/multinetlab-amsterdam/projects/tree/master/activity_network_project_2023
