# Supplementary material for: The relationship between pathological brain activity and functional network connectivity in glioma patients
Source: J Neurooncol. 2024 Feb 3;166(3):523–33. doi: 10.1007/s11060-024-04577-7 (PMC10876827; doi:10.1007/s11060-024-04577-7)
Supplement: Supplementary file 1 — Supplementary file1 (DOCX 135 KB) [file 11060_2024_4577_MOESM1_ESM.docx]

**Supplementary materials**

**The relationship between pathological brain activity and functional network connectivity in glioma patients**

Mona L.M. Zimmermann^1,2,3^*, Lucas C. Breedt^1,2^, Eduarda G. Z. Centeno^1,4^, Jaap C. Reijneveld^5^, Fernando A.N. Santos^1,6^, Cornelis J. Stam^7^, Marike R. van Lingen^1,2,3^, Menno M. Schoonheim^1,2^, Arjan Hillebrand^2,7^, Linda Douw^1,2,3^

Author affiliations:

1. Anatomy and Neurosciences, Amsterdam UMC location Vrije Universiteit Amsterdam

2. Amsterdam Neuroscience, Amsterdam UMC location Vrije Universiteit Amsterdam

3. Cancer Center Amsterdam, Amsterdam UMC location Vrije Universiteit Amsterdam

4. Univ. Bordeaux, CNRS, IMN, UMR 5293

5. Department of Neurology, Stichting Epilepsie Instellingen Nederland

6. Dutch Institute for Emergent Phenomena (DIEP), Institute for Advanced Studies, University of Amsterdam

7. Clinical Neurophysiology and MEG Center, Amsterdam UMC location Vrije Universiteit Amsterdam

Correspondence to: Mona L.M. Zimmermann

m.l.m.zimmermann@amsterdamumc.nl

**Table S1 Overview of studies that published on (partly) the same data as the current study**

| **Authors (Year)** | **Title** |
| --- | --- |
| Douw et al. (2010) [1] | Epilepsy is related to theta band brain connectivity and network topology in brain tumor patients |
| van Dellen et al. (2012)[2] | MEG Network Differences between Low- and High-Grade Glioma Related to Epilepsy and Cognition |
| van Dellen et al. (2012)[3] | Connectivity in MEG resting-state networks increases after resective surgery for low-grade glioma and correlates with improved cognitive performance. |
| Carbo et al. (2017)[4] | Dynamic hub load predicts cognitive decline after resective neurosurgery |
| Derks et al. (2018)[5] | Oscillatory brain activity associates with neuroligin-3 expression and predicts progression free survival in patients with diffuse glioma. |
| Derks et al. (2019)[6] | Understanding cognitive functioning in glioma patients: The relevance of IDH-mutation status and functional connectivity |
| Belgers et al. (2020)[7] | Postoperative oscillatory brain activity as an add-on prognostic marker in diffuse glioma. |
| Numan et al. (2021)[8] | Non-invasively measured brain activity and radiological progression in diffuse glioma |
| Derks et al. (2021)[9] | Understanding Global Brain Network Alterations in Glioma Patients |
| van Lingen et al. (2023) [10] | The longitudinal relation between executive functioning and multilayer network topology in glioma patients |
| *Note.* Table adapted from Röttgering et al. (2023)[11] | |

| ***Table S2* Network characteristics (density 30%) in the investigated areas of patients and their comparison to whole brain characteristics of HCs** | | | | | | | | | |
| --- | --- | --- | --- | --- | --- | --- | --- | --- | --- |
| Measure | Delta | | | Theta | | | Lower Alpha | | |
|  | 30% | | | 30% | | | 30% | | |
|  | mean  (SD) | *U* | *p*  *(p_FDR_)* | mean  (SD) | *U* | *p*  *(p_FDR_)* | mean  (SD) | *U* | *p*  *(p_FDR_)* |
| Clustering Coefficient |  | | |  | | |  | | |
| Peritumoral Area | 0.364  (1.156) | 2920 | <0.001  (<0.001**) | 0.246  (1.466) | 2169 | 0.551  (0.551) | 0.155  (1.350) | 2212 | 0.423  (0.507) |
| Contralateral Homologue Area | 0.335  (1.159) | 2906 | <0.001  (<0.001**) | 0.297  (1.320) | 2295 | 0.231  (0.277) | 0.225  (1.209) | 2670 | 0.003  (0.005*) |
| Rest of the brain | 0.399  (1.231) | 3948 | <0.001  (<0.001**) | 0.351  (1.377) | 3711 | <0.001  (<0.001**) | 0.295  (1.290) | 3390 | <0.001  (<0.001**) |
| HCs | 0  (0.992) |  |  | 0  (0.992) |  |  | 0  (0.992) |  |  |
| Eigenvector Centrality |  |  |  |  |  |  |  |  |  |
| Peritumoral Area | 0.128  (1.305) | 2206 | 0.439  (0.717) | -0.017  (1.098) | 2163 | 0.570  (0.717) | -0.036  (1.030) | 1944 | 0.637  (0.717) |
| Contralateral Homologue Area | 0.035  (1.079) | 2020 | 0.913  (0.966) | 0.072  (1.097) | 1972 | 0.735  (0.966) | -0.025  (1.046) | 2034 | 0.966  (0.966) |
| Rest of the brain | -0.058  (1.106) | 1582 | <0.001  (<0.001**) | -0.023  (1.122) | 2208 | 0.157  (0.188) | -0.019  (1.127) | 2308 | 0.310  (0.310) |
| HCs | 0  (0.992) |  |  | 0  (0.992) |  |  | 0  (0.992) |  |  |
|  |  |  |  |  |  |  |  |  |  |
| *Note.* * indicates p <0.05, ** indicates p<0.001; HCs = Healthy controls; SD = Standard Deviation; *U* = U statistic of the Mann-Whitney U test; *p_FDR_ =* False Discovery Rate adjusted p-value. For network characteristics p-values were corrected for the different frequencies and densities. The means of the measures were calculated with the values standardized on the regional means and SD of HCs (dev). Therefore, the mean of HCs is 0 and the SD around 1. | | | | | | | | | |

| ***Table S3* Linear Mixed Model with offset_dev_ as dependent and EC_dev_ and CC_dev_ as independent variables for the peritumoral and contralateral homologue areas in patients** | | | | | |
| --- | --- | --- | --- | --- | --- |
| Frequency,  Density | Variable | Coefficient [CI] | *Z* | *p* | *p_FDR_* |
| Peritumoral Area |  |  |  |  |  |
| Delta |  |  |  |  |  |
| 20% | Intercept | 1.573 [1.276, 1.871] | 10.358 | < 0.005 |  |
|  | EC_dev_ | 0.073 [-0.005, 0.151] | 1.832 | 0.067 | 0.200 |
|  | CC_dev_ | 0.022 [-0.057, 0.102] | 0.549 | 0.583 | 0.777 |
| 30% | Intercept | 1.546 [1.249, 1.843] | 10.192 | <0.001 |  |
|  | EC_dev_ | 0.078 [-0.001, 0.156] | 1.945 | 0.052 | 0.200 |
|  | CC_dev_ | 0.083 [0.003, 0.163] | 2.022 | 0.043 | 0.200 |
| Theta |  |  |  |  |  |
| 20% | Intercept | 1.591 [1.291, 1.891] | 10.397 | <0.005 |  |
|  | EC_dev_ | 0.089 [0.007, 0.171] | 2.121 | 0.034 | 0.200 |
|  | CC_dev_ | 0.013 [-0.062, 0.087] | 0.329 | 0.742 | 0.815 |
| 30% | Intercept | 1.586 [1.286, 1.886] | 10.372 | <0.005 |  |
|  | EC_dev_ | 0.074 [-0.01, 0.157] | 1.728 | 0.084 | 0.201 |
|  | CC_dev_ | 0.035 [-0.041, 0.11] | 0.899 | 0.369 | 0.632 |
| Lower Alpha |  |  |  |  |  |
| 20% | Intercept | 1.600 [1.301, 1.9] | 10.486 | <0.005 |  |
|  | EC_dev_ | -0.011 [-0.104, 0.082] | -0.234 | 0.815 | 0.815 |
|  | CC_dev_ | -0.029 [-0.112, 0.053] | -0.698 | 0.485 | 0.727 |
| 30% | Intercept | 1.588 [1.289, 1.887] | 10.415 | <0.005 |  |
|  | EC_dev_ | -0.049 [-0.139, 0.042] | -1.054 | 0.292 | 0.584 |
|  | CC_dev_ | 0.013 [ -0.069, 0.095] | 0.309 | 0.757 | 0.815 |
| Contralateral Homologue Area |  |  |  |  |  |
| Delta |  |  |  |  |  |
| 20% | Intercept | 0.398 [0.155, 0.642] | 3.203 | 0.001 |  |
|  | EC_dev_ | -0.021[-0.087,0.044] | -0.633 | 0.527 | 0.584 |
|  | CC_dev_ | -0.038 [-0.097, 0.022] | -1.241 | 0.214 | 0.409 |
| 30% | Intercept | 0.392 [0.148, 0.635] | 3.155 | 0.002 |  |
|  | EC_dev_ | -0.03 [-0.095, 0.035] | -0.902 | 0.367 | 0.489 |
|  | CC_dev_ | -0.019 [-0.077, 0.04] | -0.62 | 0.535 | 0.584 |
| Theta |  |  |  |  |  |
| 20% | Intercept | 0.373 [0.135, 0.612] | 3.064 | 0.002 |  |
|  | EC_dev_ | 0.08 [0.018, 0.612] | 2.532 | 0.011 | 0.045* |
|  | CC_dev_ | 0.032 [-0.025, 0.09] | 1.097 | 0.273 | 0.409 |
| 30% | Intercept | 0.379 [0.14, 0.618] | 3.108 | 0.002 |  |
|  | EC_dev_ | 0.079 [0.018, 0.141] | 2.537 | 0.011 | 0.045* |
|  | CC_dev_ | 0.009 [-0.05, 0.068] | 0.303 | 0.762 | 0.540 |
| Lower Alpha |  |  |  |  |  |
| 20% | Intercept | 0.364 [0.119, 0.609] | 2.915 | 0.004 |  |
|  | EC_dev_ | 0.041 [-0.027, 0.109] | 1.175 | 0.240 | 0.409 |
|  | CC_dev_ | 0.053 [-0.008, 0.113] | 1.711 | 0.087 | 0.260 |
| 30% | Intercept | 0.353 [0.108, 0.598] | 2.827 | 0.005 |  |
|  | EC_dev_ | 0.043 [-0.025, 0.111] | 1.242 | 0.214 | 0.409 |
|  | CC_dev_ | 0.084 [0.024, 0.145] | 2.736 | 0.006 | 0.045* |
| *Note.* * indicates p <0.05, ** indicates p<0.001; A random intercept was fitted for participants; CI = Confidence interval for coefficient; *p_FDR_* = False Discovery Rate adjusted p-value. The p-values were corrected for the different frequency bands and densities, separately for the two areas. Only the independent variables were included in this correction. | | | | | |

**Tumor masks and area definitions**

To define the peritumoral area, masks were either manually drawn in, slice by slice [LD], on post-gadolinium T1-weighted and FLAIR anatomical images [12], or automatically segmented using a neural network algorithm [13]. Subsequently, for every subject, we calculated the volume overlap of the tumor masks with the regions of the BNA using FSL (version 6.0.5.1) to determine which BNA regions contained tumor. We then calculated the percentage overlap between the tumor and every region by dividing the normal volume of a region with the volume of the tumor mask within that region. Next, we plotted all percentage volume overlaps of all regions of all subjects in a histogram. This helped us to determine the percentage overlap that was the minimum overlap still commonly represented in patients. This minimum overlap was 12%. Therefore, regions were defined to be part of the peritumoral area when at least 12% of the region’s volume overlapped with the tumor mask.

**Magnetoencephalography**

Participants underwent a 5-minute eyes-closed resting-state MEG in supine position, using a 306-channel Elekta Neuromag Oy MEG (Helsinki, Finland) system in a magnetically shielded room, with a sampling frequency of 1250Hz and online 0.1Hz high pass and 410Hz antialiasing filters. We used cross-validation signal space separation (SSS), after which raw data were visually inspected and malfunctioning channels were excluded [LD]. To remove artefacts offline, these channels were removed before applying the temporal extension of Signal Space Separation in MaxFilter software (Elekta NeuroMag Oy, version 2.2.15) to the raw data. The signal was subsequently filtered between 0.5-45Hz using a single-pass finite impulse response filter in MaxFilter. We used a 3D digitizer (Fastrak; Polhelmus, Colchester, VT, USA) to digitize 4 or 5 head position indicator coils, as well as the scalp surface and nose to enable co-registration of the MEG data to the patients’ anatomical MRIs using surface matching. Subsequently, a scalar beamformer implementation (Elekta Neuromag Oy, version 2.1.28) source-reconstructed broadband (0.5-45Hz) MEG time series to the centroids of the 210 cortical regions of the Brainnetome atlas [14-16]. We then selected 15 epochs for patients and eight epochs for HCs (each 4 * 4096 samples of 3.27s) for further analysis. These were the smallest number of good quality epochs available for any subject in the cohorts.

**Fitting Oscillations & One Over F (FOOOF)**

The Fitting Oscillations & One Over F (FOOOF) toolbox was used to estimate the offset by fitting the non-oscillatory part of the power spectrum using the exponential function L: L = b – log(k + Fx) [17]. The parameter b is the offset, the power of the lowest frequency of the power spectrum; k is the bending of the aperiodic part and was set to 0; F is a vector containing all frequencies, and x is the slope of the aperiodic part.

**Functional network thresholding**

There is no agreed standard pipeline to threshold functional networks as of yet. We decided to use a proportional threshold by keeping only the n% strongest links. We used multiple densities (20%, 30%) to investigate whether results would replicate across densities and therefore be robust. At first, we additionally calculated a threshold of 10%. However, networks only containing the 10% strongest links showed many unconnected, isolated nodes, not allowing us to investigate our graph theoretical measures of interest. Therefore, we decided not to go further with a 10% threshold in our final analysis. We used the same thresholding procedure for all subjects (patients and HCs) in the study. Furthermore, we decided to choose 30% as the highest density, as we wanted to avoid false positives in our networks. A density higher than 30% might then result in a too highly connected network with a more random network topology. Also, previous neuroimaging studies have similarly used a 30% threshold for this reason, further supporting this thresholding choice [18-20].

**Within-subject relationships using Pearson correlations**

As a second approach to the within-subject analysis, we correlated regional offset_dev_ with CC_dev_ and EC_dev_ using Pearson’s correlation in every participant. To obtain two group-level within-subject correlation values, we calculated the weighted mean of the correlations by first z-transforming the correlations using Fisher’s z- transform and then weighting these by the number of regions that were used in the initial correlation and finally taking the mean. To see whether this correlation was significant at the group-level, we used a Wilcoxon signed rank test against 0, in which we inputted each participant’s Fisher z-transformed correlation value. To then test whether the relationship in patients differed from that in HCs, we used a Mann-Whitney U test with Fisher z-transformed correlations as input. Results from these analysis were similar to the LMM approach: offset_dev_ related negatively to lower alpha CC_dev_ in the rest of the brain of patients, but only for a 20% density after FDR correction (Table S11). This significantly differed from HCs for 30% density, who again did not show a relationship between offset_dev_ and CC_dev_ for the lower alpha band (Table S11). HCs again showed a positive relationship between delta offset_dev_ and CC_dev_, but this did not differ significantly from patients.

The relationship between offset_dev_ and EC_dev_ for the lower alpha band was similar to the LMMs when using Pearson’s correlations for patients with offset_dev_ relating negatively to EC_dev_ (Table S11). For HCs, the positive relationship between offset_dev_ and delta EC_dev_, was similar as well and was now significantly different from that in patients (Table S11).

| ***Table S4*** **Network characteristics in the investigated areas of patients and the comparison between peritumoral and contralateral homologue areas** | | | | | | | | |
| --- | --- | --- | --- | --- | --- | --- | --- | --- |
| Measure,  Area,  Comparison | Delta | | | Theta | | Lower Alpha | | |
|  | 20% | 30% | | 20% | 30% | 20% | | 30% |
| Clustering Coefficient |  | | |  | |  | | |
| Peritumoral Area  (mean (SD)) | 0.370  (1.179) | 0.364  (1.156) | | 0.257  (1.447) | 0.246  (1.466) | 0.129  (1.323) | | 0.155  (1.350) |
| Contralateral Homologue Area  (mean (SD)) | 0.276  (1.137) | 0.335  (1.159) | | 0.289  (1.301) | 0.297  (1.320) | 0.233  (1.227) | | 0.225  (1.209) |
| Comparison Peritumoral and Contralateral Homologue Area  (*U, (p, p_FDR_))* | 975  (0.306, 0.611) | 1134  (0.975, 0.975) | | 1123  (0.920, 0.975) | 1132  (0.965, 0.975) | 862  (0.084, 0.501) | | 955  (0.250, 0.611) |
|  |  | |  | |  | |  | |
| Eigenvector Centrality |  | |  | |  | |  | |
| Peritumoral Area  (mean (SD)) | 0.139  (1.313) | 0.128  (1.305) | | -0.010  (1.114) | -0.017  (1.098) | -0.092  (1.044) | | -0.036  (1.030) |
| Contralateral Homologue Area  (mean (SD)) | 0.037  (1.059) | 0.035  (1.079) | | 0.058  (1.109) | 0.072  (1.097) | -0.031  (1.079) | | -0.025  (1.046) |
| Comparison Peritumoral and Contralateral Homologue Area  (*U (p, p_FDR_))* | 1075  (0.689, 0.940) | 1080  (0.712, 0.940) | | 1100  (0.808, 0.940) | 1127  (0.940, 0.940) | 978  (0.315, 0.940) | | 1043  (0.549, 0.940) |
| *Note.* * indicates p <0.05, ** indicates p<0.001; SD = Standard Deviation; *U* = U statistic of the Mann-Whitney U test; *p_FDR_ =* False Discovery Rate adjusted p-value. P-values were corrected for the different frequency bands and densities. The means of the measures were calculated with the values standardized on the regional means and SD of HCs (dev). | | | | | | | | |

**Post-hoc subgroup analyses**

In order to better understand the surprising relationship between CC_dev_ and offset_dev_, we performed post-hoc analyses within patient subgroups according to molecular tumor types, namely IDH-wildtype glioblastoma, IDH-mutant, 1p/19q-codeleted, and IDH-mutant, 1p/19q non-codeleted glioma patients. We classified 75 subjects with regards to IDH-mutation and 1p/19q deletion status. For most subjects recruited after 2016, this data was readily available as the IDH-mutation and 1p19q deletion status were determined in standard clinical care, based on the 2016 and 2021 classification of human tumors [21, 22]. For a subgroup of subjects (N = 29), we were able to extrapolate the status based on the initial pathology report and clinical data. Ten patients had an IDH-mutant astrocytoma and we therefore classified them as having IDH-mutant, 1p/19q non-codeleted gliomas. Five patients had an astrocytoma with a survival of over 3 years. These were classified as IDH-mutant, 1p/19q non-codeleted gliomas. Two patients had an astrocytoma grade two or three while being IDH-wildtypes and a survival of less than a year, these were therefore classified as IDH-wildtype gliomas. Eight patients had a GBM, so we extrapolated that these similarly had IDH-wildtype gliomas. Four patients had an oligodendroglioma. These were classified as having an IDH-mutated 1p/19q-codeleted glioma. All subgroups showed higher peritumoral activity in comparison to HCs (Table S5), while only patients with IDH-mutant, 1p/19q-codeleted and non-codeleted gliomas showed higher activity throughout the brain. Network characteristics per subgroup were similar to results from the entire group (Table S6, Table S7). The post-hoc tests of within-subject analysis focused on the lower alpha band, based on the interesting relationships between CC_dev_ and offset_dev_ that we found in the group-level analyses of this study. The negative correlation between offset_dev_ and CC_dev_ was significant in patients with an IDH-wildtype glioblastoma (only for one density) for LMMs (Table S12), but not when performing the correlation analysis (Table S13). The relationship between offset_dev_ and EC_dev_ was negative for IDH-wildtype glioblastoma and IDH-mutant, 1p/19q-codeleted gliomas, in both the LMM and correlation analyses (Table S12, S13). Finally, we again did not find a significant relationship between peritumoral offset and these correlations for either of the molecular subtypes, further indicating that the observed effects are widespread and independent of activity differences directly around the tumor (Table S14).

## Activity-dependence of regional correlations

Finally, we explored whether the level of peritumoral activity would drive the relationships between distant activity and network connectivity, which could indicate global effects of local pathological activity. Using Pearson’s correlations, we tested whether peritumoral offset_dev_ was associated with the correlations (within-subject correlations, Supplementary materials) between activity and regional network characteristics throughout the brain. We used the mean offset_dev_ across all peritumoral regions and the mean of the three peritumoral regions with the highest offset_dev_ to be maximally sensitive to any effects. We found no significant associations between peritumoral offset_dev_ and the within-patient associations between regional offset_dev_ and either CC_dev_ or EC_dev_ (Table S14), suggesting that the negative correlations between activity and connectivity throughout the brain were not dependent on the level of peritumoral activity. Aberrant activity around the tumor is likely not the primary driver of network-activity correlations further away in this cohort but that more complex mechanisms may be at play.

## Post-hoc covariate analysis

In a post-hoc test we investigated the effect of three covariates on the within-subject relationship between CC, EC and activity. We conducted the mixed model analysis three more times, including one of the following three covariates as an extra variable: handedness (left, right, ambidexter), tumor hemisphere (left, right, bilateral) and whether the tumor is located in the dominant hemisphere (based on handedness, dominant, non-dominant). For handedness, we included 83 patients as we were not able to find out the handedness for one subject. For tumor hemisphere, all subjects were included. For the covariate location in the dominant hemisphere we included 75 patients (bilateral tumor was not counted). We determined the dominant hemisphere based on the tumor location and the handedness of the subjects: if the tumor was located contralateral to the handedness, we called it dominant, otherwise non-dominant. We found no significant effect of any of the three covariates and the results were not immensely affected by the inclusion of these covariates (see Tables S18 , S19 and S20). Only when including the tumor hemisphere as covariate, the negative relationship between EC and activity did not stay significant when thresholding the network at 20% density.

| ***Table S5* Offset in the glioma subtypes for the investigated areas including comparison to HCs (whole brain) and between peritumoral and homologue areas** | | | | | | | | | | | |
| --- | --- | --- | --- | --- | --- | --- | --- | --- | --- | --- | --- |
| Subtype | Peritumoral Area | | | Contralateral Homologue Area | | | Rest of the brain | | | Comparison Peritumoral Area to Homologue Area | |
|  | mean (SD) | *U* | *p*  *(p_FDR)_* | mean (SD) | *U* | *p*  *(p_FDR_)* | mean (SD) | *U* | *p*  *(p_FDR_)* | *Z* | *p* |
| IDH-wildtype glioblastoma | 1.841  (1.579) | 1309 | <0.0001  (<0.001**) | 0.292  (1.132) | 864 | 0.199  (0.199) | 0.339 (1.349) | 1129 | 0.071  (0.071) | 7 | <0.001** |
| IDH- mutant, 1p/19q non-codeleted | 1.666  (1.622) | 1189 | <0.0001  (<0.001**) | 0.266  (1.166) | 863 | 0.106  (0.106) | 0.403  (1.174) | 1125 | 0.002  (0.048*) | 5 | <0.001** |
| IDH-mutant, 1p/19q- codeleted | 1.217  (1.302) | 565 | <0.001  (0.001*) | 0.493  (1.020) | 518 | 0.004  (0.013*) | 0.409  (1.317) | 696 | 0.032  (0.048*) | 18 | 0.206 |
| *Note.* * indicates p <0.05, ** indicates p<0.001; SD = Standard Deviation; U = U statistic of the Mann-Whitney U test; p_FDR_ = False Discovery Rate adjusted p-value. P-values were corrected for the different areas. | | | | | | | | | | | |

| ***Table S6* Network characteristics (for 20% density) in the rest of the brain of patients with different glioma subtypes including comparison to HCs (whole brain)** | | | | | | | | | | |
| --- | --- | --- | --- | --- | --- | --- | --- | --- | --- | --- |
| Measure,  Subgroup | Delta | | | Theta | | | | Lower Alpha | | |
|  | 20% | | | | 20% | | | 20% | | |
|  | mean (SD) | *U* | *p*  *(p_FDR_)* | | mean (SD) | *U* | *p*  *(p_FDR_)* | mean (SD) | *U* | *p*  *(p_FDR_)* |
| Clustering Coefficient |  | | | | | | | | | |
| IDH-wildtype glioblastoma | 0.470  (1.269) | 1523 | <0.001  (<0.001**) | | 0.398  (1.557) | 1292 | 0.001  (0.002*) | 0.230  (1.242) | 1164 | 0.036  (0.043*) |
| IDH-mutant, 1p/19q non-codeleted | 0.330  (1.199) | 1325 | <0.001  (<0.001**) | | 0.357  (1.380) | 1325 | <0.001  (<0.001**) | 0.226  (1.204) | 1135 | 0.013  (0.016*) |
| IDH-mutant, 1p/19q codeleted | 0.117  (1.176) | 635 | 0.160  (0.160) | | 0.243  (1.296) | 700 | 0.028  (0.074) | 0.191  (1.250) | 650 | 0.021  (0.074) |
| HCs | 0  (0.992) |  |  | | 0  (0.992) |  |  | 0  (0.992) |  |  |
| Eigenvector Centrality |  | | | | | | | | | |
| IDH-wildtype glioblastoma | -0.098  (1.111) | 504 | <0.001  (0.002*) | | -0.043  (1.138) | 673 | 0.041  (0.083) | -0.011  (1.116) | 812 | 0.387  (0.464) |
| IDH-mutant, 1p/19q non-codeleted | -0.050  (1.087) | 551 | 0.008  (0.022*) | | -0.023  (1.129) | 772 | 0.471  (0.707) | -0.003  (1.101) | 852 | 0.989  (0.989) |
| IDH-mutant, 1p/19q codeleted | -0.047  (1.068) | 379 | 0.093  (0.169) | | -0.035  (1.089) | 483 | 0.672  (0.735) | -0.042  (1.112) | 379 | 0.093  (0.169) |
| HCs | 0  (0.992) |  |  | | 0  (0.992) |  |  | 0  (0.992) |  |  |
| *Note*. * indicates p <0.05, ** indicates p<0.001; SD = Standard Deviation; U = U statistic of the Mann-Whitney U test; *p_FDR_* = False Discovery Rate adjusted p-value. P-values were corrected for the different frequency bands and densities. The means of the measures were calculated with the values standardized on the regional means and SD of HCs (dev). Therefore, for HC the mean is 0 and SD around 1. | | | | | | | | | | |

| ***Table 7* Network characteristics (for 30% density) in the rest of the brain of patients with different glioma subtypes including comparison to HCs (whole brain)** | | | | | | | | | | | | |
| --- | --- | --- | --- | --- | --- | --- | --- | --- | --- | --- | --- | --- |
| Measure,  Subgroup | Delta | | | | Theta | | | | Lower Alpha | | | |
|  | 30% | | | 30% | | | | | | 30% | | |
|  | mean (SD) | *U* | *p*  *(p_FDR_)* | mean (SD) | | *U* | *p*  *(p_FDR_)* | | | mean  (SD) | *U* | *p*  *(p_FDR_)* |
| Clustering Coefficient |  | | | | | | |  |  |  |  |  |
| IDH-wildtype glioblastoma | 0.579  (1.282) | 1539 | <0.001  (<0.001*) | 0.431  (1.509) | | 1329 | <0.001  (<0.001**) | | | 0.207  (1.203) | 1150 | 0.048  (0.048*) |
| IDH-mutant, 1p/19q non-codeleted | 0.372  (1.194) | 1350 | <0.001  (<0.001**) | 0.374  (1.330) | | 1350 | <0.001  (<0.001**) | | | 0.213  (1.155) | 1102 | 0.029  (0.029*) |
| IDH-mutant, 1p/19q codeleted | 0.230  (1.195) | 709 | 0.021  (0.074) | 0.269  (1.303) | | 691 | 0.037  (0.074) | | | 0.191  (1.179) | 655 | 0.099  (0.135) |
| HCs | 0  (0.992) |  |  | 0  (0.992) | |  |  | | | 0  (0.992) |  |  |
| Eigenvector Centrality |  | | | | | | |  |  |  |  |  |
| IDH-wildtype glioblastoma | -0.089  (1.135) | 481 | <0.001  (0.002*) | -0.035  (1.143) | | 688 | 0.056  (0.084) | | | -0.003  (1.107) | 907 | 0.949  (0.949) |
| IDH-mutant, 1p/19q non-codeleted | -0.049  (1.098) | 538 | 0.005  (0.022*) | -0.017  (1.133) | | 801 | 0.643  (0.771) | | | 0.004  (1.087) | 942 | 0.439  (0.707) |
| IDH-mutant, 1p/19q codeleted | -0.046  (1.087) | 344 | 0.035  (0.169) | -0.021  (1.090) | | 490 | 0.735  (0.735) | | | -0.026  (1.090) | 387 | 0.113  (0.169) |
| HCs | 0  (0.992) |  |  | 0  (0.992) | |  |  | | | 0  (0.992) |  |  |
| *Note*. * indicates p <0.05, ** indicates p<0.001; SD = Standard Deviation; U = U statistic of the Mann-Whitney U test; *p_FDR_* = False Discovery Rate adjusted p-value. P-values were corrected for the different frequency bands and densities. The means of the measures were calculated with the values standardized on the regional means and SD of HCs (dev). Therefore, for HC the mean is 0 and SD around 1. | | | | | | | | | | | | |

| ***Table S8*** **Spin-test results for patients and HCs** | | | | | | |
| --- | --- | --- | --- | --- | --- | --- |
| Measure,  Group | Delta | | Theta | | Lower Alpha | |
|  | 20% | 30% | 20% | 30% | 20% | 30% |
| CC and offset |  | |  | |  | |
| Patients (*r* [*p_binom_*]) | 0.562  [0,<0.001]** | 0.613  [0<0.001]** | 0.598  [0, <0.001]** | 0.629  [0, <0.001]** | 0.579  [0, <0.001]** | 0.608  [0, <0.001]** |
| HCs (*r* [*p_binom_*]) | 0.757  [0, <0.001]** | 0.769  [0, <0.001]** | 0.705  [0, <0.001]** | 0.715  [0, <0.001]** | 0.653  [0, <0.001]** | 0.703  [0, <0.001]** |
| EC and offset |  | |  | |  | |
| Patients (*r* [*p_binom_*]) | 0.124  [0.522, 0.549] | 0.099  [0.591, 0.618] | 0.405  [0.028, 0.038]* | 0.339  [0.061, 0.075] | 0.212  [0.308, 0.333] | 0.227  [0.269, 0.295] |
| HCs (*r* [*p_binom_*]) | 0.145  [0.402, 0.429] | 0.119  [0.481, 509] | 0.353  [0.019, 0.027]* | 0.326  [0.023, 0.033]* | 0.293  [0.192, 0.214] | 0.291  [0.190, 0.214] |
|  |  |  |  |  |  |  |
| *Note.* * indicates p <0.05, ** indicates p<0.001; *r* = Pearson’s correlation; *p_binom_* = binomial confidence interval for the p-value. | | | | | | |

| ***Table S9* Linear Mixed Model with offset_de_ as dependent and EC_dev_ and CC_dev_ as independent variables including the interaction between patients and HCs to test potential differences** | | | | | |
| --- | --- | --- | --- | --- | --- |
| Frequency, Density | Variable | Coefficient [CI] | *Z* | *p* | *p_FDR_* |
| Delta |  |  |  |  |  |
| 20% | Intercept | 0 [-0,171, 0.171] | 0 | 1 |  |
|  | EC_dev_ | 0.048 [0.032, 0.065] | 5.710 | <0.001 | <0.001** |
|  | Group_patients_ x EC_dev_ | -0.043 [-0.064, -0.022] | -3.934 | <0.001 | <0.001** |
|  | CC_dev_ | 0.023 [0.005, 0.040] | 2.588 | 0.009 | 0.023* |
|  | Group_patients_ x CC_dev_ | -0.012 [-0.033, 0.01] | -1.075 | 0.282 | 0.424 |
| 30% | Intercept | 0 [-0,171, 0.171] | 0 | 1 |  |
|  | EC_dev_ | 0.047 [0.031, 0.063] | 5.596 | <0.001 | <0.001** |
|  | Group_patients_ x EC_dev_ | -0.048 [-0.07, -0.027] | -4.478 | <0.001 | <0.001** |
|  | CC_dev_ | 0.027 [0.010, 0.044] | 3.115 | 0.002 | 0.006* |
|  | Group_patients_ x CC_dev_ | -0.014 [-0.035, 0.007] | -1.284 | 0.199 | 0.319 |
| Theta |  |  |  |  |  |
| 20% | Intercept | 0 [-0,171, 0.171] | 0 | 1 |  |
|  | EC_dev_ | -0.008 [-0.025, 0.008] | -0.983 | 0.326 | 0.434 |
|  | Group_patients_ x EC_dev_ | 0.022 [0, 0.043] | 1.995 | 0.046 | 0.100 |
|  | CC_dev_ | 0.005 [-0.012, 0.022] | 0.553 | 0.581 | 0.682 |
|  | Group_patients_ x CC_dev_ | 0.002 [-0.019, 0.023] | 0.164 | 0.869 | 0.869 |
| 30% | Intercept | 0 [-0,171, 0.171] | 0 | 1 |  |
|  | EC_dev_ | -0.008 [-0.025, 0.008] | -0.999 | 0.318 | 0.434 |
|  | Group_patients_ x EC_dev_ | 0.019 [-0.002, 0.04] | 1.77 | 0.076 | 0.141 |
|  | CC_dev_ | 0.016 [-0.001, 0.033] | 1.795 | 0.073 | 0.141 |
|  | Group_patients_ x CC_dev_ | -0.008 [-0.03, 0.013] | -0.745 | 0.456 | 0.576 |
| Lower Alpha |  |  |  |  |  |
| 20% | Intercept | 0 [-0,171, 0.171] | 0 | 1 |  |
|  | EC_dev_ | -0.004 [-0.020, 0.013] | -0.424 | 0.672 | 0.701 |
|  | Group_patients_ x EC_dev_ | -0.053 [-0.074, -0.032] | -5.436 | <0.001 | <0.001** |
|  | CC_dev_ | 0.005[-0.013, 0.022] | 0.529 | 0.597 | 0.682 |
|  | Group_patients_ x CC_dev_ | -0.030 [-0.051, -0.008] | -2.717 | 0.007 | 0.018* |
| 30% | Intercept | 0 [-0,171, 0.171] | 0 | 1 |  |
|  | EC_dev_ | -0.004 [-0.020, 0.013] | -0.436 | 0.663 | 0.701 |
|  | Group_patients_ x EC_dev_ | -0.053 [-0.074, -0.032] | -4.883 | <0.001 | <0.001** |
|  | CC_dev_ | 0.016 [-0,002, 0.033] | 1.719 | 0.086 | 0.147 |
|  | Group_patients_ x CC_dev_ | -0.059 [-0.081, -0.037] | -5.321 | <0.001 | <0.001** |
| *Note.* * indicates p <0.05, ** indicates p<0.001; A random intercept was fitted for participants; CI = Confidence interval for coefficient; *p_FDR_* = False Discovery Rate adjusted p-value. The p-values were corrected for the different frequency bands and densities. Only the independent variables were included in this correction. | | | | | |

| ***Table S10* Linear Mixed Model with offset_dev_ as dependent and EC_dev_ and CC_dev_ as independent variables for the whole brain of HCs** | | | | | |
| --- | --- | --- | --- | --- | --- |
| Frequency, Density | Variable | Coefficient [CI] | *Z* | *p* | *p_FDR_* |
| Delta |  |  |  |  |  |
| 20% | Intercept | 0 [-0,147, 0.147] | 0 | 1 |  |
|  | EC_dev_ | 0.048 [0.034, 0.062] | 6.677 | <0.001 | <0.001** |
|  | CC_dev_ | 0.023 [0.008, 0.037] | 3.026 | 0.002 | 0.007* |
| 30% | Intercept | 0 [-0,147, 0.147] | 0 | 1 |  |
|  | EC_dev_ | 0.047 [0.033, 0.061] | 6.543 | <0.001 | <0.001** |
|  | CC_dev_ | 0.027 [0.013, 0.042] | 3.642 | <0.001 | 0.001* |
| Theta |  |  |  |  |  |
| 20% | Intercept | 0 [-0,147, 0.147] | 0 | 1 |  |
|  | EC_dev_ | -0.008 [-0.023, 0.006] | -1.148 | 0.251 | 0.377 |
|  | CC_dev_ | 0.005 [-0.01, 0.02] | 0.645 | 0.518 | 0.622 |
| 30% | Intercept | 0 [-0,147, 0.147] | 0 | 1 |  |
|  | EC_dev_ | -0.008 [-0.023, 0.006] | -1.166 | 0.243 | 0.377 |
|  | CC_dev_ | 0.016 [0.001, 0.031] | 2.096 | 0.036 | 0.087 |
| Lower Alpha |  |  |  |  |  |
| 20% | Intercept | 0 [-0,147, 0.147] | 0 | 1 |  |
|  | EC_dev_ | -0.004 [-0.018, 0.011] | -0.494 | 0.622 | 0.622 |
|  | CC_dev_ | 0.005 [-0.01, 0.02] | 0.616 | 0.538 | 0.622 |
| 30% | Intercept | 0 [-0,147, 0.147] | 0 | 1 |  |
|  | EC_dev_ | -0.004 [-0.018, 0.01] | -0.508 | 0.611 | 0.622 |
|  | CC_dev_ | 0.016 [0, 0.031] | 2.003 | 0.045 | 0.090 |
| *Note.* * indicates p <0.05, ** indicates p<0.001; A random intercept was fitted for participants; CI = Confidence interval for coefficient; *p_FDR_* = False Discovery Rate adjusted p-value. The p-values were corrected for the different frequency bands and densities. Only the independent variables were included in this correction. | | | | | |

| ***Table S11* Within-subject correlation results for patients (rest of the brain) and HCs (whole brain) and their group comparison** | | | | | | | | | | |
| --- | --- | --- | --- | --- | --- | --- | --- | --- | --- | --- |
| Measure,  Group | Delta | | | Theta | | | | Lower Alpha | | |
|  | 20% | 30% | | 20% | | 30% | | 20% | | 30% |
| CC_dev_ and offset_dev_ |  | | |  | | | |  | | |
| Patients rest of the brain (*r* (*p*, *p_FDR_*)) | 0.032  (0.789, 0.789) | 0.006  (0.721, 0.789) | | 0.022  (0.099, 0.179) | | 0.019  (0.119, 0.179) | | -0.035  (0.043, 0.130) | | -0.049  (0.007, 0.046*) |
| HCs (*r* (*p, p_FDR_*)) | 0.029  (0.014, 0.037*) | 0.030  (0.019, 0.037*) | | 0.014  (0.195, 0.233) | | 0.028  (0.017, 0.037*) | | 0.008  (0.419, 0.419) | | 0.018  (0.144, 0.216) |
| Group comparison (*U* (*p, p_FDR_))* | 2222  (0.174, 0.280) | 2232  (0.187, 0.280) | | 2671  (0.664, 0.681) | | 2459  (0.681, 0.681) | | 2072  (0.049, 0.149) | | 1818  (0.003, 0.017*) |
|  |  | | |  | | | |  | | |
| EC_dev_ and offset_dev_ |  | | |  | | | |  | | |
| Patients rest of the brain (*r* (*p, p_FDR_*)) | 0.002  (0.643, 0.643) | -0.004  (0.489, 0.587) | | 0.021  (0.225, 0.397) | | 0.017  (0.265, 0.397) | | -0.065  (0.009, 0.041*) | | -0.058  (0.014, 0.041*) |
| HCs (*r* (*p, p_FDR_*)) | 0.066  (<0.001, <0.001**) | 0.063  (<0.001, <0.001**) | | 0.001  (0.653, 0.986) | | -0.001  (0.771, 0.986) | | -0.007  (0.986, 0.986) | | -0.008  (0.928, 0.986) |
| Group comparison (*U* (*p, p_FDR_))* | 1717  (<0.001, 0.004*) | 1756  (0.001, 0.003*) | | 2685  (0.624, 0.624) | | 2714  (0.544, 0.624) | | 2113  (0.072, 0.124) | | 2128  (0.082, 0.124) |
|  |  | |  | |  | |  |  |  | |
| *Notes.* * indicates p <0.05, ** indicates p<0.001; r = Pearson’s correlation; *p_FDR_ =* False Discovery Rate adjusted p-value. The p-values were corrected for the different frequency bands and densities. | | | | | | | | | | |

| ***Table S12* Linear Mixed Model with offset_dev_ as dependent variable and lower alpha EC_dev_ and CC_dev_ as independent variables for the different subtypes of glioma (rest of the brain)** | | | | | | |
| --- | --- | --- | --- | --- | --- | --- |
| Frequency, Density | Variable | Coefficient [CI] | Standardized Coefficient  (betas) | *Z* | *p* | *p_FDR_* |
| IDH-wt glioblastoma | | | | | | |
| Lower Alpha |  |  |  |  |  |  |
| 20% | Intercept | 0.349 [0.063, 0.635] |  | 2.393 | 0.017 |  |
|  | EC_dev_ | -0.118 [-0.144, -0.092] | -0.098 | -8.812 | <0.001 | <0.001** |
|  | CC_dev_ | -0.021 [-0.046, 0.005] | -0.019 | -1.579 | 0.114 | 0.114 |
| 30% | Intercept | 0.357 [0.072, 0.642] |  | 2.452 | 0.014 |  |
|  | EC_dev_ | -0.101 [-0.127, -0.075] | -0.083 | -7.646 | <0.001 | <0.001** |
|  | CC_dev_ | -0.058 [-0.084, -0.032] | -0.051 | -4.363 | <0.001 | <0.001** |
| IDH-mutant, 1p19q non-codeleted | | | | | | |
| Lower Alpha |  |  |  |  |  |  |
| 20% | Intercept | 0.402 [0.135, 0.669] |  | 2.954 | 0.003 |  |
|  | ECdev | 0.008 [-0.016, 0.031] | 0.007 | 0.659 | 0.509 | 0.547 |
|  | CCdev | 0.007 [-0.16, 0.03] | 0.007 | 0.602 | 0.547 | 0.547 |
| 30% | Intercept | 0.136 [0.134, 0.668] |  | 2.947 | 0.003 |  |
|  | ECdev | 0.017 [-0.007, 0.04] | 0.015 | 1.38 | 0.167 | 0.547 |
|  | CCdev | 0.012 [-0.012, 0.036] | 0.012 | 0.951 | 0.342 | 0.547 |
| IDH-mutant, 1p19q codeleted | | | | | | |
| Lower Alpha |  |  |  |  |  |  |
| 20% | Intercept | 0.452 [0.071, 0.834] |  | 2.325 | 0.020 |  |
|  | EC_dev_ | -0.066 [-0.102, -0.031] | -0.056 | -3.647 | <0.001 | <0.001** |
|  | CC_dev_ | -0.018 [-0.051, 0.015] | -0.017 | -1.069 | 0.285 | 0.285 |
| 30% | Intercept | 0.456 [0.076, 0.837] |  | 2351 | 0.019 |  |
|  | EC_dev_ | -0.069 [-0.105, -0.034] | -0.057 | -3.838 | <0.001 | <0.001** |
|  | CC_dev_ | -0.035 [-0.07, -0.001] | -0.032 | -1.991 | 0.047 | 0.062 |
| *Note.* * indicates p <0.05, ** indicates p<0.001; A random intercept was fitted for participants; CI = Confidence interval for coefficient; *p_FDR_* = False Discovery Rate adjusted p-value. The p-values were corrected for the two densities. Only the independent variables were included in this correction. | | | | | | |

| ***Table S13* Within-subject correlation results for the different glioma subgroups (for the lower alpha band)** | | | | |
| --- | --- | --- | --- | --- |
| Subtype | CC_dev_ and offset_dev_ | | EC_dev_ and offset_dev_ | |
|  | 20% | 30% | 20% | 30% |
| IDH-wildtype glioblastoma  (*r* (*p, p_FDR_*)) | -0.0345  (0.382, 0.382) | -0.061  (0.100, 0.200) | -0.100  (0.019, 0.029*) | -0.090  (0.029, 0.029*) |
| IDH-mutant, 1p/19q non-codeleted  (*r* (*p*, *p_FDR_*)) | 0.012  (0.779, 0.849) | 0.010  (0.849, 0.849) | 0.012  (0.831, 0.831) | 0.021  (0.582, 0.831) |
| IDH-mutant, 1p/19q co-deleted  (*r* (*p*, *p_FDR_*)) | -0.055  (0.159, 0.159) | -0.070  (0.079, 0.159) | -0.101  (0.020, 0.020*) | -0.098  (0.008, 0,016*) |
| *Note.* * indicates p <0.05, ** indicates p<0.001; r = Pearson’s correlation; *p_FDR_ =* False Discovery Rate adjusted p-value. The p-values were corrected for the two densities. | | | | |

| ***Table S14* Correlations between peritumoral offset and associations in the rest of the brain** | | | | | | | | |
| --- | --- | --- | --- | --- | --- | --- | --- | --- |
| Subtype | Peritumoral Area all regions | | | | Peritumoral Area 3 highest activity regions | | | |
|  | CC_dev_ and offset_dev_  Lower Alpha | | EC_dev_ and offset_dev_  Lower Alpha | | CC_dev_ and offset_dev_  Lower Alpha | | EC_dev_ and offset_dev_  Lower Alpha | |
|  | 20% | 30% | 20% | 30% | 20% | 30% | 20% | 30% |
| Patients whole group  (*r (p, p_FDR_))* | -0.027  (0.829, 0.829) | -0.065  (0.601, 0.829) | -0.058  (0.638, 0.638) | -0.076  (0.542, 0.638) | 0.003  (0.979, 0.979) | -0.008  (0.947, 0.979) | 0.029  (0.813, 0.874) | 0.019  (0.874, 0.874) |
| IDH-wildtype glioblastoma  (*r* (*p*, *p_FDR_*)) | 0.063  (0.769, 0.944) | 0.015  (0.943, 0.943) | -0.222  (0.296, 0.338) | -0.204  (0.338, 0.338) | 0.129  (0.545, 0.579) | 0.119  (0.579, 0.579) | -0.134  (0.532, 0.606) | -0.111  (0.606, 0.606) |
| IDH-mutant, 1p/19q non-codeleted  (*r* (*p, p_FDR_*)) | -0.203  (0.353, 0.353) | -0.209  (0.339, 0.353) | -0.055  (0.803, 0.803) | -0.108  (0.622, 0.803) | -0.198  (0.366, 0.366) | -0.199  (0.361, 0.366) | -0.059  (0.787, 0.682) | -0.090  (0.682, 0.787) |
| IDH-mutant, 1p/19q-codeleted  (*r* (*p, p_FDR_*)) | -0.003  (0.993, 0.993) | -0.182  (0.591, 0.993) | 0.138  (0.685, 0.692) | 0.135  (0.692, 0.692) | -0.123  (0.717, 0.717) | -0.303  (0.363, 0.717) | 0.145  (0.670, 0.739) | 0.114  (0.739, 0.739) |
| *Notes.* * indicates p <0.05, ** indicates p<0.001; r = Pearson’s correlation; *p_FDR_ =* False Discovery Rate adjusted p-value. The p-values were corrected for the two densities. | | | | | | | | |

| ***Table*** ***S15* Linear mixed model with offset_dev_ as dependent and EC_dev_ and CC_dev_ as independent variables for the rest of the brain of patients using the AAL atlas** | | | | | |
| --- | --- | --- | --- | --- | --- |
| Frequency, Density | Variable | Coefficient [CI] | *Z* | *p* | *p_FDR_* |
| Delta |  |  |  |  |  |
| 20% | Intercept | 0.315 [0.158, 0.471] | 3.945 | <0.001 |  |
|  | EC_dev_ | 0.023 [-0.003, 0.049] | 1.744 | 0.081 | 0.139 |
|  | CC_dev_ | 0.039 [0.015, 0.063] | 3.176 | 0.001 | 0.024* |
| 30% | Intercept | 0.316 [0.160, 0.472] | 3.964 | <0.001 |  |
|  | EC_dev_ | 0.13[-0.013, 0.039] | 0.952 | 0.341 | 0.409 |
|  | CC_dev_ | 0.23[-0.001, 0.048] | 1.860 | 0.063 | 0.126 |
| Theta |  |  |  |  |  |
| 20% | Intercept | 0.318 [0.162, 0.475] | 3.985 | <0.001 |  |
|  | EC_dev_ | 0.016 [-0.010, 0.042] | 1.227 | 0.220 | 0.293 |
|  | CC_dev_ | 0.010 [-0.014, 0.035] | 0.843 | 0.399 | 0.435 |
| 30% | Intercept | 0.316 [0.159, 0.472] | 3.948 | <0.001 |  |
|  | EC_dev_ | 0.009 [-0.016, 0.035] | 0.712 | 0.447 | 0.447 |
|  | CC_dev_ | 0.020[-0.006, 0.045] | 1.521 | 0.128 | 0.192 |
| Lower Alpha |  |  |  |  |  |
| 20% | Intercept | 0.322 [0.166, 0.479] | 4.038 | <0.001 |  |
|  | EC_dev_ | -0.035 [-0.061, -0.009] | -2.651 | 0.008 | 0.024* |
|  | CC_dev_ | -0.025 [-0.050, -0.001] | -2.017 | 0.044 | 0.106 |
| 30% | Intercept | 0.323 [0.167, 0.480] | 4.052 | <0.001 |  |
|  | EC_dev_ | -0.036 [-0.062, -0.011] | -2.794 | 0.005 | 0.024* |
|  | CC_dev_ | -0.034 [-0.059, -0.009] | -2.699 | 0.007 | 0.024* |
|  |  |  |  |  |  |

*Note.* * indicates p <0.05, ** indicates p<0.001; A random intercept was fitted for participants; CI = Confidence interval for coefficient; Std = Standardized; *p_FDR_* = False Discovery Rate adjusted p-value. The p-values were corrected for the different frequency bands and densities. Only the independent variables were included in this correction.

| ***Table S16* Linear Mixed Model with offset_de_ as dependent and EC_dev_ and CC_dev_ as independent variables for the AAL atlas, including the interaction between patients and HCs to test potential differences** | | | | | |
| --- | --- | --- | --- | --- | --- |
| Frequency, Density | Variable | Coefficient [CI] | *Z* | *p* | *p_FDR_* |
| Delta |  |  |  |  |  |
| 20% | Intercept | 0 [-0,145, 0.145] | 0 | 1 |  |
|  | EC_dev_ | 0.050 [0.026, 0.073] | 4.148 | <0.001 | <0.001** |
|  | Group_patients_ x EC_dev_ | -0.027 [-0.063, 0.009] | -1.462 | 0.144 | 0.304 |
|  | CC_dev_ | -0.015 [-0.039, 0.009] | -1.199 | 0.230 | 0.425 |
|  | Group_patients_ x CC_dev_ | 0.054 [0.018, 0.089] | 2.985 | 0.003 | 0.024* |
| 30% | Intercept | 0 [-0,145, 0.145] | 0 | 1 |  |
|  | EC_dev_ | 0.050 [0.027, 0.073] | 4.201 | <0.001 | <0.001** |
|  | Group_patients_ x EC_dev_ | -0.037 [-0.073, -0.002] | -2.055 | 0.040 | 0.137 |
|  | CC_dev_ | -0.005 [-0.029, 0.019] | -0.418 | 0.676 | 0.785 |
|  | Group_patients_ x CC_dev_ | 0.029 [-0.007, 0.064] | 1.563 | 0.118 | 0.304 |
| Theta |  |  |  |  |  |
| 20% | Intercept | 0 [-0,146, 0.146] | 0 | 1 |  |
|  | EC_dev_ | -0.011 [-0.035, 0.013] | -0.915 | 0.360 | 0.576 |
|  | Group_patients_ x EC_dev_ | 0.027 [-0.009, 0.063] | 1.483 | 0.138 | 0.304 |
|  | CC_dev_ | 0.002 [-0.022, 0.026] | 0.150 | 0.881 | 0.881 |
|  | Group_patients_ x CC_dev_ | 0.009 [-0.027, 0.044] | 0.476 | 0.634 | 0.785 |
| 30% | Intercept | 0 [-0,146, 0.146] | 0 | 1 |  |
|  | EC_dev_ | -0.009 [-0.033, 0.014] | -0.754 | 0.451 | 0.637 |
|  | Group_patients_ x EC_dev_ | 0.018 [-0.017, 0.054] | 1.007 | 0.314 | 0.538 |
|  | CC_dev_ | 0.004 [-0.020, 0.028] | 0.335 | 0.738 | 0.787 |
|  | Group_patients_ x CC_dev_ | 0.016 [-0.021, 0.052] | 0.844 | 0.399 | 0.598 |
| Lower Alpha |  |  |  |  |  |
| 20% | Intercept | 0 [-0,146, 0.146] | 0 | 1 |  |
|  | EC_dev_ | -0.029 [-0.053, -0.006] | -2.432 | 0.015 | 0.06 |
|  | Group_patients_ x EC_dev_ | -0.006 [-0.056, -0.002] | -0.314 | 0.754 | 0.787 |
|  | CC_dev_ | 0.006[-0.019, 0.030] | 0.473 | 0.636 | 0.785 |
|  | Group_patients_ x CC_dev_ | -0.031[-0.068, 0.005] | -1.708 | 0.088 | 0.264 |
| 30% | Intercept | 0 [-0,146, 0.146] | 0 | 1 |  |
|  | EC_dev_ | -0.029 [-0.052, 0.006] | -2.442 | 0.015 | 0.06 |
|  | Group_patients_ x EC_dev_ | -0.007 [-0.043, 0.028] | -0.403 | 0.687 | 0.785 |
|  | CC_dev_ | 0.018 [-0,007, 0.043] | 1.431 | 0.152 | 0.304 |
|  | Group_patients_ x CC_dev_ | -0.052 [-0.089, -0.016] | -2.827 | 0.005 | 0.03* |
| *Note.* * indicates p <0.05, ** indicates p<0.001; A random intercept was fitted for participants; CI = Confidence interval for coefficient; *p_FDR_* = False Discovery Rate adjusted p-value. The p-values were corrected for the different frequency bands and densities. Only the independent variables were included in this correction. | | | | | |

| ***Table S17* Within-subject correlation results for patients (rest of the brain) and HCs (whole brain) and their group comparison using the AAL atlas** | | | | | | | | | | |
| --- | --- | --- | --- | --- | --- | --- | --- | --- | --- | --- |
| Measure,  Group | Delta | | | Theta | | | | Lower Alpha | | |
|  | 20% | 30% | | 20% | | 30% | | 20% | | 30% |
| CC_dev_ and offset_dev_ |  | | |  | | | |  | | |
| Patients rest of the brain (*r* (*p*, *p_FDR_*)) | 0.036  (0.026, 0.156) | 0.020  (0.229, 0.229) | | 0.020  (0.225, 0.229) | | 0.025  (0.100, 0.183) | | -0.028  (0.122, 0.183) | | -0.035  (0.079, 0.183) |
| HCs (*r* (*p, p_FDR_*)) | -0.007  (0.026, 0.156) | 0.001  (0.229, 0.229) | | -0.002  (0.225, 0.229) | | 0.005  (0.101, 0.183) | | 0.003  (0.122, 0183) | | 0.023  (0.079, 0.183) |
| Group comparison (*U* (*p, p_FDR_))* | 2994  (0.084, 0.252) | 2761  (0.427, 0.512) | | 2722  (0.523, 0.523) | | 2775  (0.395, 0.512) | | 2259  (0.226, 0.451) | | 2025  (0.032, 0.189) |
|  |  | | |  | | | |  | | |
| EC_dev_ and offset_dev_ |  | | |  | | | |  | | |
| Patients rest of the brain (*r* (*p, p_FDR_*)) | 0.032  (0.054, 0.162) | 0.019  (0.379, 0.455) | | 0.019  (0.233, 0.455) | | 0.011  (0.497, 0.497) | | -0.029  (0.363, 0.455) | | 0.023  (0.028, 0.162) |
| HCs (*r* (*p, p_FDR_*)) | 0.059  (0.054, 0.324) | 0.062  (0.379, 0.456 | | -0.021  (0.234, 0.456) | | -0.017  (0.498, 0.498) | | -0.034  (0.363, 0.456) | | -0.034  (0.284, 0.456) |
| Group comparison (*U* (*p, p_FDR_))* | 2320  (0.333, 0.546) | 2218  (0.169, 0.507) | | 2966  (0.106, 0.507) | | 2789  (0.364, 0.546) | | 2636  (0.768, 0.818) | | 2620  (0.818, 0.818) |
|  |  | |  | |  | |  |  |  | |
| *Notes.* * indicates p <0.05, ** indicates p<0.001; r = Pearson’s correlation; *p_FDR_ =* False Discovery Rate adjusted p-value. The p-values were corrected for the different frequency bands and densities. | | | | | | | | | | |

| ***Table S18* Linear mixed model with offset_dev_ as dependent and EC_dev_ and CC_dev_ as independent variables and handedness as covariate for the rest of the brain of patients using the BNA atlas** | | | | | |
| --- | --- | --- | --- | --- | --- |
| Frequency, Density | Variable | Coefficient [CI] | *Z* | *p* | *p_FDR_* |
| Delta |  |  |  |  |  |
| 20% | Intercept | -0.357 [-1.074, 0.361] | -0.975 | 0.330 |  |
|  | Handedness[T.L] | 0.885 [-0.014, 1.785] | 1.929 | 0.054 | 0.082 |
|  | Handedness[T.R] | 0.776 [0.039, 1.513] | 2.064 | 0.039 | 0.082 |
|  | EC_dev_ | 0.001 [-0.014, 0.016] | 0.189 | 0.850 | 0.850 |
|  | CC_dev_ | 0.008 [-0.006, 0.022] | 1.175 | 0.240 | 0.274 |
| 30% | Intercept | -0.360 [-1.077, 0.357] | -0.985 | 0.325 |  |
|  | Handedness[T.L] | 0.887 [-0.012, 1.786] | 1.933 | 0.053 | 0.082 |
|  | Handedness[T.R] | 0.777 [0.041, 1.514] | 2.068 | 0.039 | 0.082 |
|  | EC_dev_ | -0.005 [-0.020, 0.010] | -0.654 | 0.513 | 0.535 |
|  | CC_dev_ | 0.011[-0.003, 0.025] | 1.572 | 0.116 | 0.155 |
| Theta |  |  |  |  |  |
| 20% | Intercept | -0.355 [-1.073, 0.363] | -0.969 | 0.333 |  |
|  | Handedness[T.L] | 0.885 [-0.015, 1.785] | 1.927 | 0.054 | 0.082 |
|  | Handedness[T.R] | 0.775 [0.037, 1.512] | 2.059 | 0.039 | 0.082 |
|  | EC_dev_ | 0.014 [-0.001, 0.028] | 1.794 | 0.073 | 0.103 |
|  | CC_dev_ | 0.007 [-0.007, 0.020] | 0.987 | 0.324 | 0.353 |
| 30% | Intercept | -0.355 [-1.073, 0.363] | -0.968 | 0.333 |  |
|  | Handedness[T.L] | 0.883 [-0.017, 1.784] | 1.922 | 0.055 | 0.082 |
|  | Handedness[T.R] | 0.774 [0.036, 1.512] | 2.055 | 0.040 | 0.082 |
|  | EC_dev_ | 0.011 [-0.004, 0.025] | 1.438 | 0.150 | 0.189 |
|  | CC_dev_ | 0.009[-0.005, 0.023] | 1.254 | 0.210 | 0.252 |
| Lower Alpha |  |  |  |  |  |
| 20% | Intercept | -0.353 [-1.070, 0.365] | -0.964 | 0.335 |  |
|  | Handedness[T.L] | 0.900 [0, 1.799] | 1.961 | 0.050 | 0.082 |
|  | Handedness[T.R] | 0.780 [0.044, 1.517] | 2.076 | 0.038 | 0.082 |
|  | EC_dev_ | -0.060 [-0.074, -0.045] | -7.913 | <0.001 | <0.001** |
|  | CC_dev_ | -0.028 [-0.041, -0.014] | -3.985 | <0.001 | <0.001** |
| 30% | Intercept | -0.351 [-1.067, 0.365] | -0.961 | 0.337 |  |
|  | Handedness[T.L] | 0.908 [0.010, 1.806] | 1.982 | 0.047 | 0.082 |
|  | Handedness[T.R] | 0.784 [0.048, 1.520] | 2.088 | 0.037 | 0.082 |
|  | EC_dev_ | -0.053 [-0.068, -0.039] | -7.130 | <0.001 | <0.001** |
|  | CC_dev_ | -0.045 [-0.059, -0.031] | -6.229 | <0.001 | <0.001** |
|  |  |  |  |  |  |

*Note.* * indicates p <0.05, ** indicates p<0.001; A random intercept was fitted for participants; CI = Confidence interval for coefficient; Std = Standardized; *p_FDR_* = False Discovery Rate adjusted p-value. The p-values were corrected for the different frequency bands and densities. Only the independent variables were included in this correction. The covariates are in reference to category ‘ambidexter’.

| ***Table*** ***S19* Linear mixed model with offset_dev_ as dependent and EC_dev_ and CC_dev_ as independent variables and the hemisphere containing the tumor as covariate for the rest of the brain of patients using the BNA atlas** | | | | | |
| --- | --- | --- | --- | --- | --- |
| Frequency, Density | Variable | Coefficient [CI] | *Z* | *p* | *p_FDR_* |
| Delta |  |  |  |  |  |
| 20% | Intercept | 0.880 [0.163, 1.598] | 2.404 | 0.016 |  |
|  | Tumor hemisphere[T.L] | -0.423 [-1.169, 0.323] | -1.111 | 0.267 | 0.327 |
|  | Tumor hemisphere [T.R] | -0.678 [-1.440, 0.085] | -1.742 | 0.081 | 0.180 |
|  | EC_dev_ | 0.005[-0.010, 0.020] | 0.681 | 0.496 | 0.518 |
|  | CC_dev_ | 0.011 [0.003, 0.025] | 1.549 | 0.121 | 0.223 |
| 30% | Intercept | 0.878 [0.161, 1.596] | 2.401 | 0.016 |  |
|  | Tumor hemisphere [T.L] | -0.423 [-1.169, 0.323] | -1.112 | 0.266 | 0.327 |
|  | Tumor hemisphere [T.R] | -0.678 [-1.440, 0.084] | -1.744 | 0.081 | 0.180 |
|  | EC_dev_ | -0.001[-0.016, 0.013] | -0.188 | 0.851 | 0.851 |
|  | CC_dev_ | 0.013[-0.001, 0.027] | 1.859 | 0.063 | 0.180 |
| Theta |  |  |  |  |  |
| 20% | Intercept | 0.881 [0.163, 1.599] | 2.405 | 0.016 |  |
|  | Tumor hemisphere [T.L] | -0.422 [-1.169, 0.325] | -1.108 | 0.268 | 0.327 |
|  | Tumor hemisphere [T.R] | -0.678 [-1.440, 0.085] | -1.741 | 0.082 | 0.180 |
|  | EC_dev_ | 0.007 [-0.016 0.028] | 1.779 | 0.075 | 0.180 |
|  | CC_dev_ | 0.531 [-0.001, 0.020] | 0.994 | 0.320 | 0.349 |
| 30% | Intercept | 0.881 [0.162, 1.599] | 2.403 | 0.016 |  |
|  | Tumor hemisphere [T.L] | -0.422 [-1.169, 0.325] | -1.108 | 0.268 | 0.327 |
|  | Tumor hemisphere [T.R] | -0.678 [-1.441, 0.085] | -1.742 | 0.082 | 0.180 |
|  | EC_dev_ | 0.011 [-0.004, 0.025] | 1.437 | 0.151 | 0.259 |
|  | CC_dev_ | 0.008 [-0.006, 0.022] | 1.124 | 0.261 | 0.327 |
| Lower Alpha |  |  |  |  |  |
| 20% | Intercept | 0.880 [0.162, 1.598] | 2.402 | 0.016 |  |
|  | Tumor hemisphere [T.L] | -0.414 [-1.161, 0.333] | -1.086 | 0.277 | 0.327 |
|  | Tumor hemisphere [T.R] | -0.667 [-1.430, 0.096] | -1.714 | 0.087 | 0.180 |
|  | EC_dev_ | -0.063 [-0.077, -0.048] | -8.351 | 0.068 | 0.180 |
|  | CC_dev_ | -0.025 [-0.038, -0.011] | -3.627 | <0.001 | <0.001** |
| 30% | Intercept | 0.879 [0.161, 1.596] | 2.400 | <0.001 |  |
|  | Tumor hemisphere [T.L] | -0.407 [-1.153, 0.340] | -1.068 | 0.286 | 0.327 |
|  | Tumor hemisphere [T.R] | -0.660 [-1.422, 0.103] | -1.695 | 0.090 | 0.180 |
|  | EC_dev_ | -0.056[-0.071 -0.042] | -7.567 | <0.001 | <0.001** |
|  | CC_dev_ | -0.044 [-0.058, -0.030] | -6.092 | <0.001 | <0.001** |
|  |  |  |  |  |  |

*Note.* * indicates p <0.05, ** indicates p<0.001; A random intercept was fitted for participants; CI = Confidence interval for coefficient; Std = Standardized; *p_FDR_* = False Discovery Rate adjusted p-value. The p-values were corrected for the different frequency bands and densities. Only the independent variables were included in this correction. The covariates are in reference to category ‘bilateral’.

| ***Table*** ***S20* Linear mixed model with offset_dev_ as dependent and EC_dev_ and CC_dev_ as independent variables for the rest of the brain of patients using the BNA atlas. The location of the tumor in the dominant hemisphere was added as a covariate.** | | | | | |
| --- | --- | --- | --- | --- | --- |
| Frequency, Density | Variable | Coefficient [CI] | *Z* | *p* | *p_FDR_* |
| Delta |  |  |  |  |  |
| 20% | Intercept | 0.507 [0.295, 0.720] | 4.684 | <0.001 |  |
|  | Tumor in dominant hemisphere [T.non-dominant] | -0.258 [-0.600, 0.083] | -1.483 | 0.138 | 0.245 |
|  | EC_dev_ | 0.001 [-0.015, 0.017] | 0.141 | 0.888 | 0.940 |
|  | CC_dev_ | 0.000 [0.014, 0.015] | 0.059 | 0.953 | 0.953 |
| 30% | Intercept | 0.506 [0.293, 0.718] | 4.670 | <0.001 |  |
|  | Tumor in dominant hemisphere [T.non-dominant] | -0.258 [-0.599, 0.083] | -1.482 | 0.138 | 0.248 |
|  | EC_dev_ | -0.006 [-0.021, 0.010] | -0.707 | 0.479 | 0.616 |
|  | CC_dev_ | 0.003 [-0.012, 0.018] | 0.422 | 0.673 | 0.808 |
| Theta |  |  |  |  |  |
| 20% | Intercept | 0.506 [0.294, 0.719] | 4.670 | <0.001 |  |
|  | Tumor in dominant hemisphere [T.non-dominant] | -0.259 [-0.601, 0.082] | -1.488 | 0.137 | 0.248 |
|  | EC_dev_ | 0.006 [-0.009, 0.021] | 0.753 | 0.451 | 0.616 |
|  | CC_dev_ | 0.006 [-0.008, 0.019] | 0.797 | 0.425 | 0.616 |
| 30% | Intercept | 0.505 [0.292, 0.717] | 4.656 | <0.001 |  |
|  | Tumor in dominant hemisphere [T.non-dominant] | -0.261 [-0.602, 0.081] | -1.494 | 0.135 | 0.248 |
|  | EC_dev_ | 0.003 [-0.012, 0.018] | 0.354 | 0.723 | 0.813 |
|  | CC_dev_ | 0.009 [-0.005, 0.023] | 1.280 | 0.201 | 0.329 |
| Lower Alpha |  |  |  |  |  |
| 20% | Intercept | 0.513 [0.301, 0.725] | 4.733 | <0.001 |  |
|  | Tumor in dominant hemisphere [T.non-dominant] | -0.258 [-0.600, 0.083] | -1.482 | 0.138 | 0.248 |
|  | EC_dev_ | -0.061 [-0.076, -0.046] | -7.775 | <0.001 | <0.001** |
|  | CC_dev_ | -0.023 [-0.037, -0.009] | -3.210 | 0.001 | 0.005* |
| 30% | Intercept | 0.519 [0.307, 0.731] | 4.794 | <0.001 |  |
|  | Tumor in dominant hemisphere [T.non-dominant] | -0.259 [-0.601, 0.082] | -1.490 | 0.136 | 0.248 |
|  | EC_dev_ | -0.054 [-0.070, -0.039] | -6.988 | <0.001 | <0.001** |
|  | CC_dev_ | -0.039 [-0.054, -0.025] | -5.294 | <0.001 | <0.001** |
|  |  |  |  |  |  |

*Note.* * indicates p <0.05, ** indicates p<0.001; A random intercept was fitted for participants; CI = Confidence interval for coefficient; Std = Standardized; *p_FDR_* = False Discovery Rate adjusted p-value. The p-values were corrected for the different frequency bands and densities. Only the independent variables were included in this correction.

**References**

1. Douw L et al (2010) Epilepsy is related to theta band brain connectivity and network topology in brain tumor patients. BMC Neuroscience. 11(1): p. 103. https://doi.org/10.1186/1471-2202-11-103

2. van Dellen E et al (2012) MEG Network Differences between Low- and High-Grade Glioma Related to Epilepsy and Cognition. PLoS ONE. 7(11): p. e50122. https://doi.org/10.1371/journal.pone.0050122

3. van Dellen E et al (2013) Connectivity in MEG resting-state networks increases after resective surgery for low-grade glioma and correlates with improved cognitive performance. NeuroImage: Clinical. 2: p. 1-7. https://doi.org/10.1016/j.nicl.2012.10.007

4. Carbo EWS et al (2017) Dynamic hub load predicts cognitive decline after resective neurosurgery. Scientific Reports. 7(1): p. 42117. https://doi.org/10.1038/srep42117

5. Derks J et al (2018) Oscillatory brain activity associates with neuroligin-3 expression and predicts progression free survival in patients with diffuse glioma. Journal of Neuro-Oncology. 140(2): p. 403-412. https://doi.org/10.1007/s11060-018-2967-5

6. Derks J et al (2019) Understanding cognitive functioning in glioma patients: The relevance of IDH‐mutation status and functional connectivity. Brain and Behavior. 9(4): p. e01204. https://doi.org/10.1002/brb3.1204

7. Belgers V et al (2020) Postoperative oscillatory brain activity as an add-on prognostic marker in diffuse glioma. Journal of Neuro-Oncology. 147(1): p. 49-58. https://doi.org/10.1007/s11060-019-03386-7

8. Numan T et al (2021) Non-invasively measured brain activity and radiological progression in diffuse glioma. Scientific Reports: p. 10. https://doi.org/10.1038/s41598-021-97818-y

9. Derks J et al (2021) Understanding Global Brain Network Alterations in Glioma Patients. Brain Connectivity. 11(10): p. 865-874. https://doi.org/10.1089/brain.2020.0801

10. van Lingen MR et al (2023) The longitudinal relation between executive functioning and multilayer network topology in glioma patients. Brain imaging and behavior, 17(4), 425–435. https://doi.org/10.1007/s11682-023-00770-w

11. Röttgering JG et al (2023) Symptom networks in glioma patients: understanding the multidimensionality of symptoms and quality of life. Journal of Cancer Survivorship. https://doi.org/10.1007/s11764-023-01355-8

12. Derks J et al (2017) Connectomic profile and clinical phenotype in newly diagnosed glioma patients. NeuroImage: Clinical. 14: p. 87-96. https://doi.org/10.1016/j.nicl.2017.01.007

13. Bouget D et al (2022) Preoperative Brain Tumor Imaging: Models and Software for Segmentation and Standardized Reporting. Frontiers in Neurology. 13: p. 18. https://doi.org/10.3389/fneur.2022.932219

14. Hillebrand A et al (2016) Direction of information flow in large-scale resting-state networks is frequency-dependent. Proceedings of the National Academy of Sciences. 113(14): p. 3867-3872. https://doi.org/10.1073/pnas.1515657113

15. Fan L et al (2016) The Human Brainnetome Atlas: A New Brain Atlas Based on Connectional Architecture. Cerebral Cortex. 26(8): p. 3508-3526. https://doi.org/10.1093/cercor/bhw157

16. Hillebrand A et al (2012) Frequency-dependent functional connectivity within resting-state networks: An atlas-based MEG beamformer solution. NeuroImage. 59(4): p. 3909-3921. https://doi.org/10.1016/j.neuroimage.2011.11.005

17. Donoghue T et al (2020) Parameterizing neural power spectra into periodic and aperiodic components. Nature Neuroscience. 23(12): p. 1655-1665. https://doi.org/10.1038/s41593-020-00744-x

18. Bassett DS, Bullmore ET (2006) Small-World Brain Networks. The Neuroscientist. 12(6): p. 512-523. https://doi.org/10.1177/1073858406293182

19. Bassett DS, Bullmore ET (2017) Small-World Brain Networks Revisited. The Neuroscientist. 23(5): p. 499-516. https://doi.org/10.1177/1073858416667720

20. Lynall ME et al (2010) Functional Connectivity and Brain Networks in Schizophrenia. Journal of Neuroscience. 30(28): p. 9477-9487. https://doi.org/10.1523/JNEUROSCI.0333-10.2010

21. Louis DN et al (2016) The 2016 World Health Organization Classification of Tumors of the Central Nervous System: a summary. Acta Neuropathologica. 131(6): p. 803-820. https://doi.org/10.1007/s00401-016-1545-1

22. Louis DN et al (2021) The 2021 WHO Classification of Tumors of the Central Nervous System: a summary. Neuro-Oncology. 23(8): p. 1231-1251. https://doi.org/10.1093/neuonc/noab106
